# Functional chromatin features are associated with structural mutations in cancer

**DOI:** 10.1186/1471-2164-15-1013

**Published:** 2014-11-23

**Authors:** Krzysztof R Grzeda, Beryl Royer-Bertrand, Koichiro Inaki, Hyunsoo Kim, Axel M Hillmer, Edison T Liu, Jeffrey H Chuang

**Affiliations:** The Jackson Laboratory for Genomic Medicine, 10 Discovery Drive, Farmington, CT 06030 USA; Department of Medical Genetics, University of Lausanne, 1005 Lausanne, Switzerland; Genome Technology and Biology, Genome Institute of Singapore, Singapore, 138672 Singapore; The Jackson Laboratory, Bar Harbor, ME 04609 USA

**Keywords:** Protein binding, Chromatin state, Structural mutations, Cancer

## Abstract

**Background:**

Structural mutations (SMs) play a major role in cancer development. In some cancers, such as breast and ovarian, DNA double-strand breaks (DSBs) occur more frequently in transcribed regions, while in other cancer types such as prostate, there is a consistent depletion of breakpoints in transcribed regions. Despite such regularity, little is understood about the mechanisms driving these effects. A few works have suggested that protein binding may be relevant, e.g. in studies of androgen receptor binding and active chromatin in specific cell types. We hypothesized that this behavior might be general, i.e. that correlation between protein-DNA binding (and open chromatin) and breakpoint locations is common across divergent cancers.

**Results:**

We investigated this hypothesis by comprehensively analyzing the relationship among 457 ENCODE protein binding ChIP-seq experiments, 125 DnaseI and 24 FAIRE experiments, and 14,600 SMs from 8 diverse cancer datasets covering 147 samples. In most cancers, including breast and ovarian, we found enrichment of protein binding and open chromatin in the vicinity of SM breakpoints at distances up to 200 kb. Furthermore, for all cancer types we observed an enhanced enrichment in regions distant from genes when compared to regions proximal to genes, suggesting that the SM-induction mechanism is independent from the bias of DSBs to occur near transcribed regions. We also observed a stronger effect for sites with more than one protein bound.

**Conclusions:**

Protein binding and open chromatin state are associated with nearby SM breakpoints in many cancer datasets. These observations suggest a consistent mechanism underlying SM locations across different cancers.

**Electronic supplementary material:**

The online version of this article (doi:10.1186/1471-2164-15-1013) contains supplementary material, which is available to authorized users.

## Background

Somatic structural mutations (SM) have long been recognized as a major player in cancer development and treatment responsiveness [1]. A classic example comes from chronic myelogenous leukemia, in which presence of a structural variation fusing the genes BCR and ABL is closely associated with susceptibility to the drug imatinib [2, 3]. By causing deletion of tumor-suppressor genes, duplicating proto-oncogenes, creating new fusion genes, or altering gene regulation, SMs may interfere with normal cell differentiation programs and lead to tumorigenesis.

SMs result from interaction and defective repair of DNA double-strand breaks (DSBs) [4, 5], usually through nonhomologous end joining [6] or microhomology-mediated end joining [4, 5]. Complex mutations may also arise through chromoplexy (a chain of balanced interchromosomal translocations involving more than two chromosomes) [7], chromothripsis (a catastrophic event involving shattering of a chromosome with subsequent joining of pieces in random order and orientation) and chromoanasynthesis (a collection of multiple interspersed copy number gains) [8]. Despite the importance of SMs in cancer, the mechanisms governing their locations are not fully understood. For example, end-joining events in cancer have only ~1 nt more homology at joined sites than expected by chance, making analysis of these events mostly uninformative and incapable of predicting where DSBs may occur on the genome scale. A few broad features correlating with SM breakpoints have been identified [5, 9, 10]. The foremost known correlate of DSBs is transcriptionally active chromatin [10], which largely coincides with other commonly reported predictors such as replication timing, GC content [5] and negative G-band staining [9].

Recent studies have suggested that the spatial structure of the genome is a factor governing the locations of SM events [1], although three-dimensional genome structure characterizations are still relatively low resolution. For example, spatial proximity of chromatin segments [11], which in some regions is regimented [12, 13], has been observed to increases the likelihood of interaction to form a new structural variation [13]. We hypothesize that such spatial proximity may be related to protein binding and transcription. This hypothesis is motivated by evidence indicating that chromatin regions are organized during interphase into “transcription factories”, in which DNA segments are looped together by specific constellations of transcription factors in a nuclear compartment [14, 15]. The relationship to protein binding is also supported by the fact that key DNA-binding proteins such as CTCF and cohesin are known to maintain vertebrate chromatin structure [16] and to separate chromatin domains [17, 18].

A few examples of either open chromatin or protein binding events influencing SM locations are also known. In B cells, a yeast I-SceI endonuclease motif was inserted into the genome to become a fixed locus for DSB induction; subsequently the induced DSBs were found to preferentially join to regions of active chromatin [10, 19]. In prostate cancer cell lines, binding of androgen receptor to DNA has been shown to determine which exons would participate in translocation, with the specific location of the DSB determined to ~10 bp precision by short sequence motifs [20].

In this paper, we demonstrate that these types of associations between protein binding/chromatin state on the one hand and SMs on the other hand are not isolated to the experimental systems where they were originally described. We perform a comprehensive analysis of 457 protein binding ChIP-seq experiments, 125 DnaseI, and 24 FAIRE experiments from the ENCODE project and multiple cancer SM callsets (breast, ovarian, head&neck, colorectal and prostate). Our results indicate that DNA-protein binding and open chromatin are widespread and common features associated with SMs.

## Methods

### Datasets

We used multiple published SM callsets, with no requirement to obtain a separate ethical approval, from a variety of types of tumors (Table 1) to analyze the relationship between binding/chromatin and breakpoint locations. We selected the datasets generated using three different pipelines to rule out the possibility of a systematic, pipeline-specific bias.

**Table 1:** **Overview of the SM callsets used in the study**

Callset	Number of samples	Number of SM events	Sequencing platform	Aligner	Original reference genome	Caller	Reference
Wellcome Trust Sanger Institute							
Breast-Stephens	24	2113	Illumina GAII 2 × 37 bp insert 500 bp	MAQ	hg18	SSAHA	[6]
Breast-NikZainal	21	1149	Illumina GAIIx 2 × 108 bp Hiseq 2000 2 × 100 bp insert <700 bp	bwa/MAQ	hg19	SSAHA	[21]
Ovarian-McBride	13	631	Illumina GA2 2 × 37 bp insert 200-500 bp	bwa	hg19	SSAHA	[22]
Broad Institute							
Colorectal-Bass	9	653	Illumina GA-II 2 × 101 bp insert 400 bp	MAQ	hg18	dRanger	[23]
Head&Neck-Stransky	2	126	Illumina GA II 2 × 101 bp insert 380-400 bp	bwa	hg19	dRanger	[24]
Prostate-Berger	7	755	Illumina GA II 2 × 101 bp insert 400 bp	MAQ	hg18	dRanger	[25]
Prostate-Baca	57	5710	Illumina GA II 2 × 101 bp insert 340 bp	bwa	hg19	dRanger	[26]
Other							
Breast-Inaki	14	3463	SOLiD long span 10 kb 2 × 36 bp	Corona Lite bwa	hg18 hg19	custom unnamed	[27, 28]

Coordinates were unified using LiftOver (http://genome.ucsc.edu/cgi-bin/hgLiftOver) to remap to the hg19 reference as needed. We analyzed all inter- and intrachromosomal SM events such that both breakpoints fall in autosomal chromosomes. To calculate odds ratio separately for each SM callset, we divided the whole genome into regions based on the distance to the nearest SM breakpoint. Positions within 50 kb from the nearest breakpoint were deemed to be “in the vicinity” of SM breakpoints, and all other positions were deemed to be “outside of the vicinity”. Formally, we defined a vicinity *C* as:


where *sm* iterates through all breakpoints reported in the Stephens SM callset, *d*(*x*, *sm*) denotes distance between *x* and *sm*, and the union is performed for all sites on autosomal chromosomes.

We downloaded peak calls from 457 protein binding ChIP-seq experiments, 125 DnaseI experiments, and 24 FAIRE experiments from the ENCODE website [29, 30]. For the odds ratio calculations for each of those datasets, we used peaks on autosomal chromosomes.

### Enrichment

In order to calculate enrichment separately near and far form genes, we used annotations of transcribed regions as downloaded from Ensembl (http://uswest.ensembl.org/). For the comparison of SM breakpoints and gene bodies, we identified the transcribed site (including intronic regions) nearest to each SM breakpoint regardless of strand/orientation. Distance to the gene was defined as the absolute distance between the SM breakpoint and the nearest transcription start or end, whichever was closer, regardless of strand and orientation; if the breakpoint was in the interior of a transcript, that distance was deemed zero. According to that definition, the regions within 60 kb of any gene were considered “near genes” and all the remaining regions were considered “far from genes”.

In order to quantify ChIP-seq and open chromatin enrichment in the vicinities of SM breakpoint, we calculated two enrichment metrics (i.e. fraction of coverage and odds ratio) for each pair of a ChIP-seq or open chromatin experiment (e.g. TAF1 in cell line GM12878 in lab HAIB) and an SM callset (e.g. Breast-Stephens).

Fraction of coverage indicates the fraction of ChIP-seq peaks falling into a certain distance range from the SM breakpoints. For example, the fraction of coverage in the 100 kb-200 kb range from breakpoints in the Breast-Stephens callset, was calculated as


where *H*_*TAF*1,*GM*12878,*HAIB*,*experiment*_ denotes a set of all genomic positions under at least one ChIP-seq peak for TAF1 in the GM12878 cell line in given *experiment* performed by the HAIB lab.

These observed values were compared against null model expectations based on the size of the vicinities:


where asterisk denotes complements to the entire autosomal genomes.

**Figure 1:**
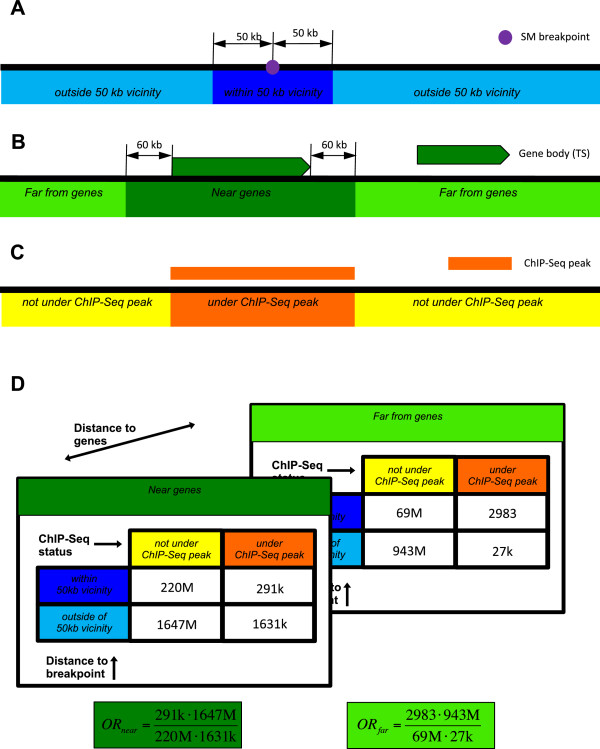
**Enrichment calculations.** Enrichment calculations are based on dividing the genome in the autosomal chromosomes according to three criteria: **A**. distance to the nearest SM breakpoint, **B**. distance to the nearest gene, and **C**. relationship to ChIP-Seq peaks. **D**. A schematic representation of a 2 × 2 × 2 contingency table with two 2 × 2 slices (“Near genes” and “Far from genes”) for calculating odds ratio separately near and far from genes.

We also calculated odds ratio (OR) as a measure of relative overrepresentation of protein binding ChIP-seq or open chromatin coverage in the vicinities around an SM breakpoint, as shown in Figure 1.

### Odds ratio statistics

In some of the protein ChIP-seq or open chromatin experiments there were only very few peaks detected, resulting in one or more of the entries in the 2 × 2 × 2 contingency table (see Figure 1D) being 0. In order to properly quantify odds ratios in the regions near and far from genes and to perform an unbiased comparisons between them, we accepted only the experiments with non-zero entries in all cells of the 2 × 2 × 2 contingency table. This procedure effectively filtered out the experiments with infinite log odds ratios in at least one distance category (near or far from genes).

Subsequently, the odds ratio were converted to their base 2 logarithms and we calculated mean and standard deviation across multiple protein ChIP-seq or open chromatin experiments. A two-tailed t-test was then used to assess how significantly the log odds ratios (or their difference) deviates from zero.

### Synergy

To test for synergistic behavior between protein binding sites, we performed calculations separately in each combination of lab and cell line where ChIP-seq experiments for at least two proteins were available. We first identified the union of ChIP-seq peaks for each protein in a given cell line × lab combination:


where *experiment* iterates through all ENCODE experiments (antibody etc.) available for a given protein, cell line and lab. This union step was necessary because even in a given cell line and lab, there may be multiple measurements for a single protein under slightly modified conditions. Subsequently, for each position in the genome we calculated the number of proteins with evidence of binding at that site


where *protein* iterates through all proteins with data available in a given cell line and lab, and […] denotes the indicator function. This allowed as to divide the genome into sets with evidence of binding different numbers of proteins


where *k* indicates number of binding proteins.

To evaluate whether sites with evidence of binding 2 proteins are more enriched near the SM breakpoints than sites with evidence of only 1 protein binding, we then calculated odds ratio:


where subscripts other than protein count have been omitted for brevity.

Finally, we calculated mean, standard deviation and *p* value (against null model of odds ratio being 1) of base 2 logarithm of those odds ratios across all cell line and lab pairs.

## Results

### Chromothriptic and chromoplectic prostate cancers display similar pattern of protein binding enrichment in the vicinity of SM breakpoints

Prostate cancers are an important model for studying SMs. Approximately half of all prostate adenocarcinomas contain a fusion of an ETS transcription factor with a nearby gene, most typically ETS-related gene (ERG) with transmembrane protease serine-2 (TMPRSS2) [26]. That fusion often arises in a chromoplectic mechanism, and such ETS-positive prostate cancers are further predisposed to have more interchromosomal rearrangements than other prostate cancers, especially near highly expressed genes. A contrasting genomic aberration in prostate cancer is deletion of CHD1 (chromodomain helicase DNA-binding protein-1), a gene involved in maintaining DNA stability. Prostate cancers with a CHD1 deletion demonstrate predominantly intrachromosomal rearrangements and are enriched for SMs in heterochromatic regions, characteristic of chromothripsis [26, 31]. Accordingly, the SM events in ETS+/CHD1wt tumors would arise mainly through chromoplexy and those in ETSwt/CHD1del would arise through chromothripsis.

We took advantage of these distinct molecular subtypes of prostate cancer to investigate common behaviors in the relationship between protein-binding sites and locations of SM breakpoints. To do so, we classified each base position in the autosomal chromosomes according to whether it was under an ENCODE ChIP-seq peak and whether it was in or outside the vicinity of an SM breakpoint (≤50 kb). In addition, we tabulated whether each base position was near (≤60 kb) or far (>60 kb) from a gene, in order to distinguish the effect of gene proximity (See Methods, Figure 1). Subsequently, for each ChIP-seq experiment, we calculated two odds ratio values, one near the genes and one far from the genes, to assess enrichment of ChIP-seq signal in the vicinity of SM breakpoints.

**Figure 2:**
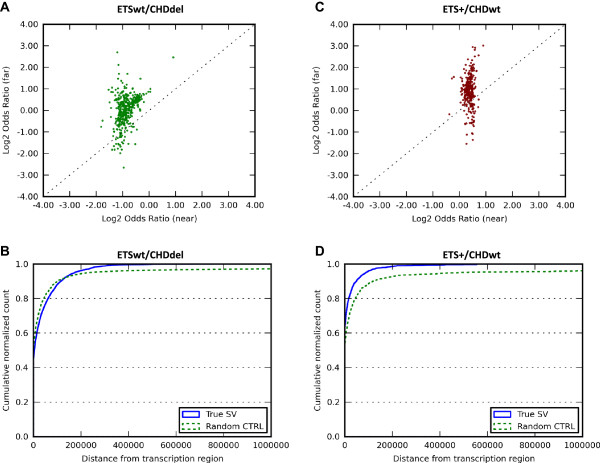
**Comparison of the ETSwt/CHD1del (left) and ETS+/CHD1wt (right) subsets of the Prostate-Baca SM callset. A** and **C**. Odds ratio values across all available ChIP-seq experiments. Each point represents a different protein binding ChIP-seq experiment, with odds ratio calculated separately near (≤60 kb) genes (horizontal axis) and far from (>60 kb) genes (vertical axis). Positive values indicate enrichment of protein binding ChIP-seq signal within 50 kb of SM breakpoints. **B** and **D**. Cumulative normalized histogram of distance between an SM breakpoint and the nearest gene for the autosomal intrachromosomal events in the SM callsets.

Figure 2A shows odds ratio values in the chromothriptic ETSwt/CHD1del prostate cancer, with each datapoint showing the behavior of a separate ChIP-seq dataset. The log odds ratio near genes is negative for most ChIP-seq sets while far from genes it is generally larger (Table 2). Most data points lie above the diagonal line, indicating that the association between protein binding sites and breakpoints is stronger far from genes than near genes (p = 3.43⋅10^-98^). The low odds ratios near genes are likely due to the fact that ETSwt/CHD1del prostate cancers avoid breakpoints near genes while many protein binding sites are fixed near gene promoters. Figure 2B, shows that in ETSwt/CHD1del prostate cancers SM breakpoints are depleted up to 100 kb from the genes.

**Table 2:** **Enrichment of protein binding and open chromatin signal in the vicinity of SM breakpoints**

	ChIP-seq log _2_OR			DnaseI log _2_OR			Faire log _2_OR		
SM callset	Near ^a^	Far ^b^	Δ ^c^			Δ ^c^			Δ ^c^
			(far-near)	Near ^a^	Far ^b^	(far-near)	Near ^a^	Far ^b^	(far-near)
Breast-Menghi	0.36 ± 0.14	0.93 ± 0.71	0.58 ± 0.69	0.27 ± 0.06	0.89 ± 0.12	0.61 ± 0.11	0.24 ± 0.23	0.89 ± 0.38	0.65 ± 0.24
	p = 7.7E-172	p = 6.1E-89	p = 1.5E-47	p = 1.9E-84	p = 1.5E-109	p = 1.4E-91	p = 2.3E-04	p = 8.1E-09	p = 7.7E-10
	n = 397	n = 397	n = 397	n = 122	n = 12	n = 122	n = 19	n = 19	n = 19
Colorectal-Bass	0.10 ± 0.16	1.06 ± 0.95	0.95 ± 0.96	0.03 ± 0.06	0.96 ± 0.21	0.93 ± 0.22	0.01 ± 0.32	1.15 ± 0.40	1.14 ± 0.46
	p = 2.0E-28	p = 3.5E-62	p = 4.9E-53	p = 7.5E-06	p = 1.5E-82	p = 1.5E-78	p = 9.3E-01	p = 3.1E-10	p = 2.3E-09
	n = 345	n = 345	n = 345	n = 122	n = 122	n = 122	n = 19	n = 19	n = 19
HeadNeck-Stransky	-0.41 ± 0.29	1.24 ± 1.35	1.65 ± 1.43	-0.27 ± 0.10	0.36 ± 0.49	0.63 ± 0.50	-0.25 ± 0.28	0.21 ± 0.87	0.46 ± 0.77
	p = 1.2E-52	p = 7.0E-31	p = 8.3E-42	p = 1.5E-58	p = 5.9E-13	p = 8.6E-27	p = 1.1E-03	p = 3.1E-01	p = 2.0E-02
	n = 217	n = 217	n = 217	n = 122	n = 122	n = 122	n = 19	n = 19	n = 19
ETSwt/CHD1del subset of Prostate-Baca	-0.85 ± 0.31	0.05 ± 0.69	0.90 ± 0.63	-0.71 ± 0.21	0.16 ± 0.28	0.86 ± 0.16	-0.40 ± 0.46	0.39 ± 0.45	0.78 ± 0.24
	p = 3.6E-189	p = 1.5E-01	p = 3.4E-98	p = 2.1E-67	p = 6.8E-09	p = 4.1E-92	p = 1.3E-03	p = 1.5E-03	p = 3.6E-11
	n = 400	n = 400	n = 400	n = 122	n = 122	n = 122	n = 19	n = 19	n = 19
ETS+/CHD1wt subset of Prostate-Baca	0.37 ± 0.14	0.92 ± 0.70	0.55 ± 0.71	0.28 ± 0.04	0.93 ± 0.17	0.65 ± 0.16	0.21 ± 0.15	0.87 ± 0.40	0.66 ± 0.32
	p = 2.1E-158	p = 5.4E-78	p = 2.3E-37	p = 8.0E-102	p = 1.0E-92	p = 1.4E-78	p = 1.5E-05	p = 2.1E-08	p = 3.9E-08
	n = 348	n = 348	n = 348	n = 122	n = 122	n = 122	n = 19	n = 19	n = 19
Ovarian-McBride	0.40 ± 0.19	1.32 ± 0.80	0.92 ± 0.80	0.30 ± 0.07	1.05 ± 0.29	0.75 ± 0.25	0.18 ± 0.23	0.99 ± 0.22	0.80 ± 0.17
	p = 1.0E-130	p = 9.3E-102	p = 2.6E-66	p = 1.8E-81	p = 8.2E-71	p = 1.1E-62	p = 2.9E-03	p = 1.1E-13	p = 7.5E-14
	n = 352	n = 352	n = 352	n = 122	n = 122	n = 122	n = 19	n = 19	n = 19
Breast-NikZainal	0.17 ± 0.12	1.24 ± 0.94	1.07 ± 0.96	0.16 ± 0.05	1.16 ± 0.15	1.00 ± 0.16	0.12 ± 0.18	1.15 ± 0.47	1.03 ± 0.36
	p = 3.2E-93	p = 1.2E-82	p = 1.4E-66	p = 1.5E-69	p = 1.5E-108	p = 4.9E-98	p = 8.6E-03	p = 2.9E-09	p = 2.1E-10
	n = 368	n = 368	n = 368	n = 122	n = 122	n = 122	n = 19	n = 19	n = 19
Prostate-Berger	-0.27 ± 0.19	0.45 ± 0.77	0.72 ± 0.77	-0.26 ± 0.16	0.39 ± 0.25	0.65 ± 0.16	-0.10 ± 0.35	0.54 ± 0.33	0.64 ± 0.32
	p = 1.7E-82	p = 1.4E-22	p = 1.7E-47	p = 1.4E-35	p = 3.8E-34	p = 2.2E-76	p = 2.2E-01	p = 1.3E-06	p = 7.4E-08
	n = 332	n = 332	n = 332	n = 122	n = 122	n = 122	n = 19	n = 19	n = 19
Prostate-Baca	-0.11 ± 0.12	0.37 ± 0.65	0.48 ± 0.62	-0.16 ± 0.08	0.44 ± 0.24	0.60 ± 0.18	-0.07 ± 0.23	0.58 ± 0.40	0.64 ± 0.24
	p = 1.4E-60	p = 2.3E-27	p = 9.7E-45	p = 9.5E-43	p = 3.6E-41	p = 1.0E-68	p = 2.3E-01	p = 6.8E-06	p = 8.5E-10
	n = 413	n = 413	n = 413	n = 122	n = 122	n = 122	n = 19	n = 19	n = 19
Breast-Stephens	0.40 ± 0.12	1.28 ± 0.68	0.88 ± 0.68	0.34 ± 0.04	1.21 ± 0.15	0.88 ± 0.13	0.27 ± 0.22	1.27 ± 0.36	1.00 ± 0.22
	p = 5.6E-210	p = 3.0E-130	p = 3.1E-85	p = 5.4E-112	p = 8.6E-112	p = 1.9E-102	p = 5.0E-05	p = 1.0E-11	p = 1.5E-13
	n = 389	n = 389	n = 389	n = 122	n = 122	n = 122	n = 19	n = 19	n = 19

Enrichment of protein ChIP-seq and two open chromatin assays (DNaseI and FAIRE) signal in all SM callsets. Data in each cell show log_2_ odds ratio (mean ± standard deviation; positive values indicate enrichment). In each table row, only those protein ChIP-seq and open chromatin experiments, that have a non-zero entry in each cell of the 2 × 2 × 2 contingency table, were used; the number of such experiments is shown as *n*. **Δ** indicates difference of log OR between the regions near and far from genes.

^a^p-value calculated against a null hypothesis of log OR being 0 near genes.

^b^p-value calculated against a null hypothesis of log OR being 0 far from genes.

^c^p-value calculated against a null hypothesis of no difference in odds ratio between near and far from genes.

Figure 2C shows odds ratio values for the chromoplectic subtype ETS+/CHD1wt. For most ChIP-seq datasets, binding is enriched in the vicinity of the breakpoints near genes and enriched even more (p = 2.28⋅10^-37^) around breakpoints far from genes. Although SM breakpoints are enriched near genes for the ETS+/CHD1wt subtype (Figure 2D), protein binding enrichment around SM breakpoints is even higher far away from genes than near genes. This enhanced effect far from genes is therefore a common behavior across the chromothriptic and chromoplectic prostate cancer subtypes.

### The pattern of protein binding enrichment in the vicinity of SM breakpoints is common across many cancers

**Figure 3:**
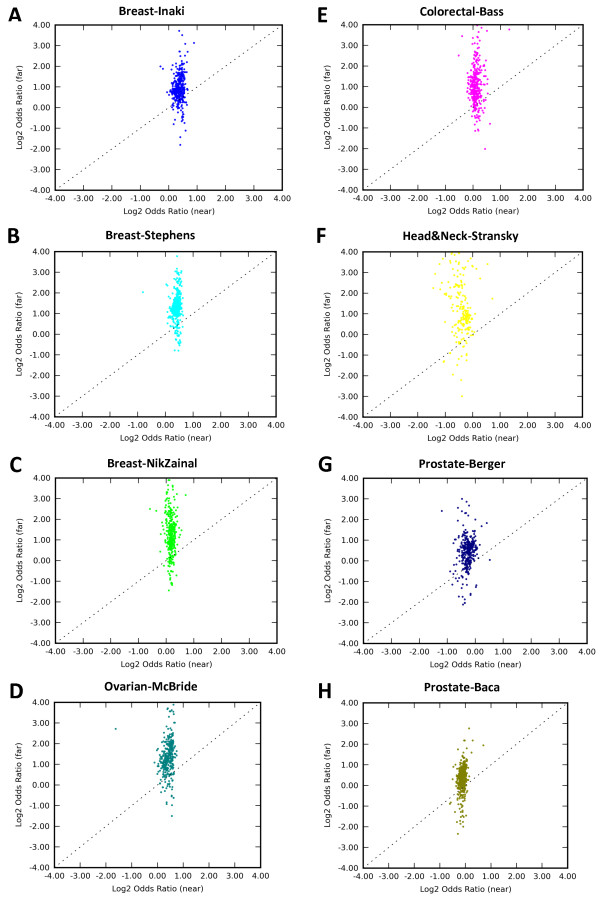
**Patterns of ChIP-seq enrichment across different cancers.** Odds ratio values across all available protein binding ChIP-seq experiments. Each point represents a different protein binding ChIP-seq experiment, with odds ratio calculated separately near (≤60 kb) genes (horizontal axis) and far from (>60 kb) genes (vertical axis). Positive values indicate enrichment of protein binding ChIP-seq signal within 50 kb of SM breakpoints. Data shown in various SM callsets: Breast-Inaki **(A)**, Breast-Stephens **(B)**, Breast-NikZainal **(C)**, Ovarian-McBride **(D)**, Colorectal-Bass **(E)**, Head&Neck-Stransky **(F)**, Prostate-Berger **(G)**, Prostate-Baca **(H)**.

Given these commonalities across prostate cancer subtypes, we hypothesized that such preferences for binding enrichment might be common in other cancers. To address this, we performed a comprehensive enrichment analysis for 8 cancer SM callsets (Table 1), encompassing 14,600 total events in breast, ovarian, colorectal, prostate and head&neck cancers. For most of the cancers (breast, ovarian and colorectal) the SM breakpoints were enriched in the gene regions (these cancers will be referred to as “genophilic”), while in some others (prostate and head&neck) they were depleted (these cancers will be referred to as “genophobic”) or showed high variability, in agreement with previous reports [5] (Additional file 1). In every cancer we studied, the odds ratio of protein binding ChIP-seq enrichment in the SM vicinity was higher far from genes than near genes. Average enrichment metrics are summarized in Table 2 and the complete data for all protein binding sets are visualized in Figure 3. Furthermore, the relationship between odds ratio in the regions near and far from genes remained true in every cancer when inter- and intrachromosomal events were considered separately (Additional file 2).

**Figure 4:**
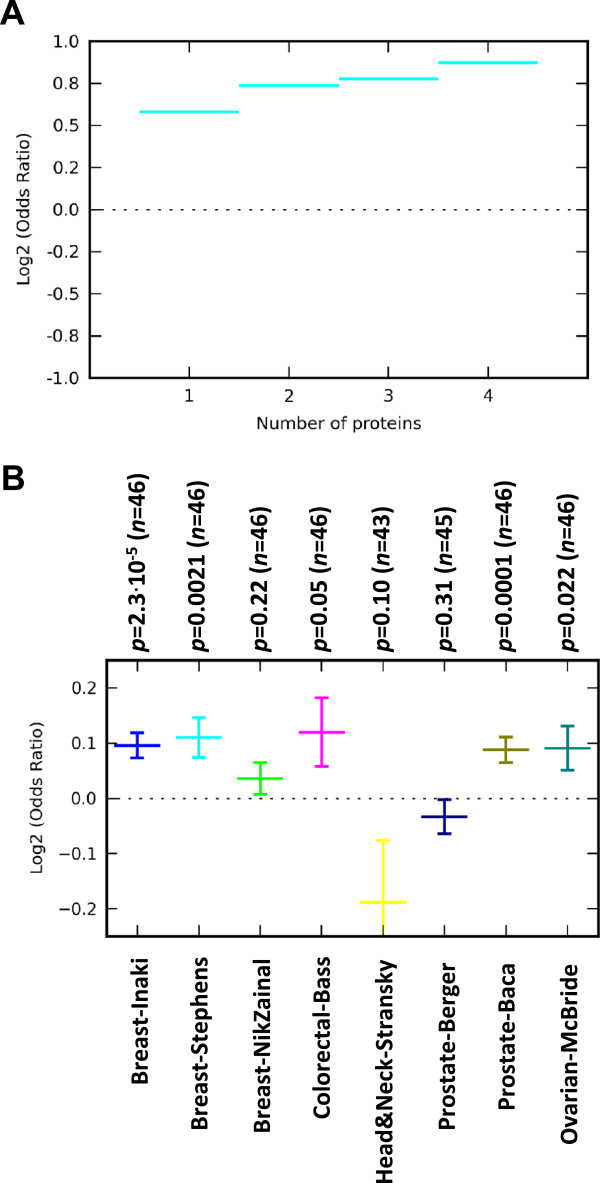
**Synergy between multiple proteins. A**. Odds ratio for enrichment of protein bindings in the vicinity of SM breakpoints (within 50 kb) as a function of the number of bound proteins (in the Breast-Stephens callset). Protein binding data are from the ENCODE A549 cell line as measured in the HAIB lab. **B**. Comparison of sites with evidence of binding exactly 2 proteins vs. exactly 1 protein. The vertical axis shows log_2_ odds ratio (mean ± standard error), where mean and error are calculated based on the number of cell line × lab combinations with available data (*n*) for at least 2 proteins. Statistical significance (*p*) is with respect to a null model of log-odds ratio being 0 (horizontal dotted line).

We next inquired whether sites with multiple evidence of proteins binding might have an even stronger association with breakpoints. To address this question, we calculated odds ratios separately for sites with one bound protein, two bound proteins, three proteins and so on with respect to the null hypothesis that sites are distributed randomly along the genome. We observed that odds ratio tends to increase with the number of bound proteins, as shown in a representative example in Figure 4A for the A549 cell line from the HAIB lab. To assess in a systematic way whether the sites binding 2 proteins are indeed more enriched in the vicinity of SM breakpoints than sites binding just 1 protein, we calculated log_10_ odds ratio for all cell line and lab combinations with ChIP-seq data available for at least 2 proteins. The results, visualized in Figure 4B, demonstrate that sites binding exactly two proteins were more enriched within 50 kb of breakpoints than sites binding exactly one in the genophilic cancers: Breast-Inaki (*p* = 2.3⋅10^-5^), Breast-Stephens (*p* = 0.0021), Breast-NikZainal (*p* = 0.22), Ovarian-McBride (*p* = 0.022) and Colorectal-Bass (*p* = 0.05).

### Chromatin state is also predictive of SM breakpoints

**Figure 5:**
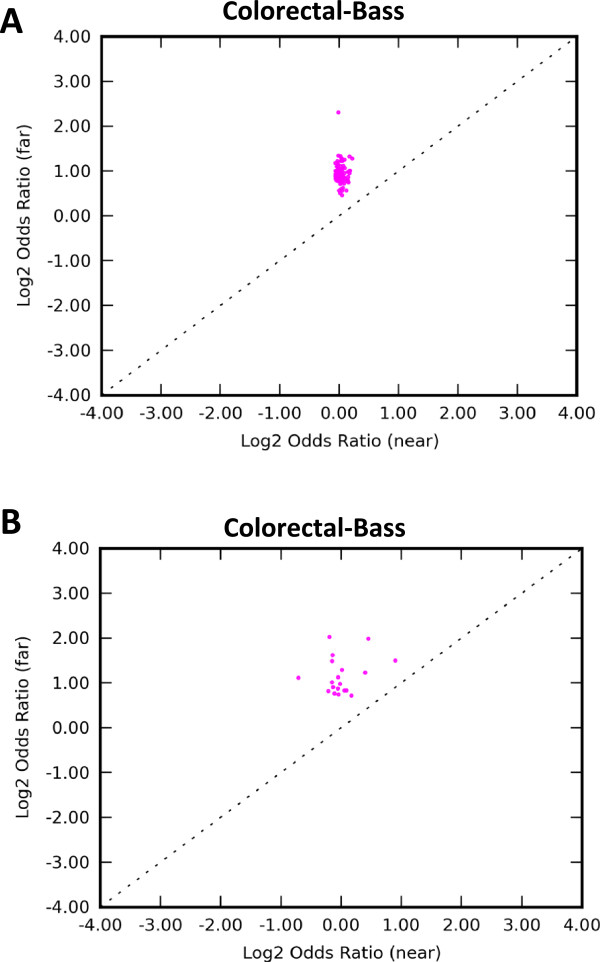
**Patterns of open chromatin enrichment.** Odds ratio values across all available DnaseI **(A)** and FAIRE **(B)** experiments in the Colorectal-Bass callset. Each point represents a different open chromatin experiment, with odds ratio calculated separately near (≤60 kb) genes (horizontal axis) and far from (>60 kb) genes (vertical axis). Positive values indicate enrichment of open chromatin signal within 50 kb of SM breakpoints.

Our results (Additional file 3) reveal no strong protein-specific pattern in regard to protein binding enrichment in the vicinity of SM breakpoint. This suggests that SM breakpoints might be associated with a higher level feature such as open chromatin. To gain deeper insight, we analyzed evidence for open chromatin in the vicinity of SM breakpoints, using DnaseI and FAIRE assay data sets. In every cancer studied, the odds ratio of open chromatin enrichment in the vicinity of breakpoints was higher far from genes than near genes, similar to the protein-binding patterns (Table 2). Moreover, these findings also remained true when inter- and intrachromosomal events were considered separately (Additional file 2). More specifically, in the genophilic cancers open chromatin was enriched in the vicinity of SM breakpoints both near and far from genes (Figure 5 shows an example), in both DnaseI and FAIRE assays. In the remaining (genophobic) cancers, the enrichment log odds ratio was negative near genes while positive far from genes.

**Figure 6:**
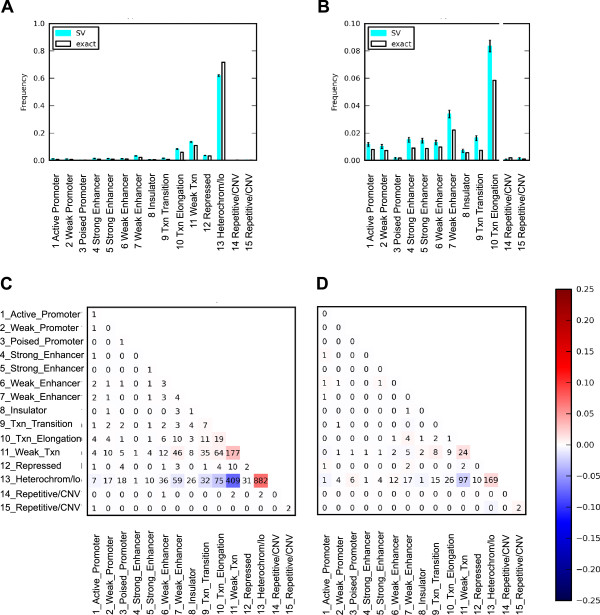
**Distribution of functional chromatin state at the breakpoints. A**. Histogram of chromatin state (Broad-GM12878) at the breakpoints in the Breast-Stephens SM callset. Teal and white bars indicate observed vs. expected values, respectively. Error bars indicate binomial standard error. **B**. Zoom-in view of chromatin state histogram for selected states. **C** and **D**. Correlations between chromatin state (Broad-H1HESC) for pairs of breakpoints from the same SM in the Breast-Stephens SM callset. The numbers indicate counts of SM events with their breakpoints in the two labeled states. The color scale indicates difference between the observed frequency of SM events and the expectation if breakpoints were independent, presented on a linear scale (eg. observed frequency of 30% vs. expected of 20% maps to 0.10 on the scale). **C**. Both inter- and intrachromosomal events. **D**. Interchromosomal events only.

To get a more detailed picture of the relationship between breakpoints and functional chromatin state, we also analyzed functional chromatin state directly at the breakpoints. The chromatin state annotations were previously predicted from chromatin marks such as histone methylation and acetylation along the genome in 9 ENCODE cell types [32]. Consistent with our protein binding analysis above, we observed enrichment of breakpoints in states associated with both transcribed regions and enhancers. As an example, Figure 6A and B shows chromatin state enrichment in the GM12878 cell line for breakpoints in the Breast-Stephens SM callset. This shows enrichment for breakpoints in promoter, enhancer and transcribed states, with a depletion in heterochromatin. More broadly, breakpoints were consistently enriched in the transcription states (states 9–11) in the Breast-Inaki, Breast-Stephens, Breast-NikZainal and Ovarian-McBride SM callsets, in all 9 cell lines (Additional file 4). Similar enrichment was observed in those SM callsets in the enhancer regions (states 4–7) in all 9 cell lines, except for Breast-NikZainal in the NHLF cell line. Furthermore, breakpoints were also consistently enriched in the promoter regions (states 1–3) in Breast-Inaki, Breast-Stephens and Ovarian-McBride, except for Ovarian-McBride in the HUVEC cell line, while no consistent enrichment pattern was observed in Breast-NikZainal. Conversely, breakpoints were consistently depleted in the heterochromatin regions (state 13) in Breast-Inaki, Breast-Stephens, Breast-NikZainal and Ovarian-McBride in all 9 cell lines. In general, we observed preferences for enhancers and promoters and avoidance of heterochromatin for all cancers except prostate and head&neck.

In addition, we observed biases in the paired states of the two breakpoints of each SM event. We calculated the frequencies of state pairs and compared against a null model assuming random matching (Figure 6C). SM events with both breakpoints in the same state, such as transcriptional elongation (state 10), weak transcribed (state 11) and heterochromatin/low signal (state 13) were enriched as compared to the behavior of each state individually. This is in part because breakpoint pairs are predominantly local and intrachromosomal, and the genome contains large blocks of both heterochromatic and transcribed regions. Nevertheless, when only interchromosomal events were considered, the pattern of enrichment remained similar, notably with enrichment for both ends in heterochromatin (state 13) and depletion in events with one end in weak transcription (state 11) and the other in heterochromatin (state 13) (Figure 6D). This suggests that during processes in which structural mutations arise, there are interactions between the breakpoint sites influenced by their chromatin state.

### Distance considerations

We also were interested in how far away from the SM breakpoints the enrichment of protein binding and open chromatin state would extend, to ascertain the robustness of our findings to distance thresholds. We therefore calculated enrichment of ChIP-seq signal and open chromatin assays in the vicinity of SM breakpoints as a function of distance. To do so, we divided the genome into disjoint regions parameterized by the distance. We then calculated two enrichment metrics in each of such regions: fraction of ChIP-seq coverage falling into each given bin, and odds ratio.

**Figure 7:**
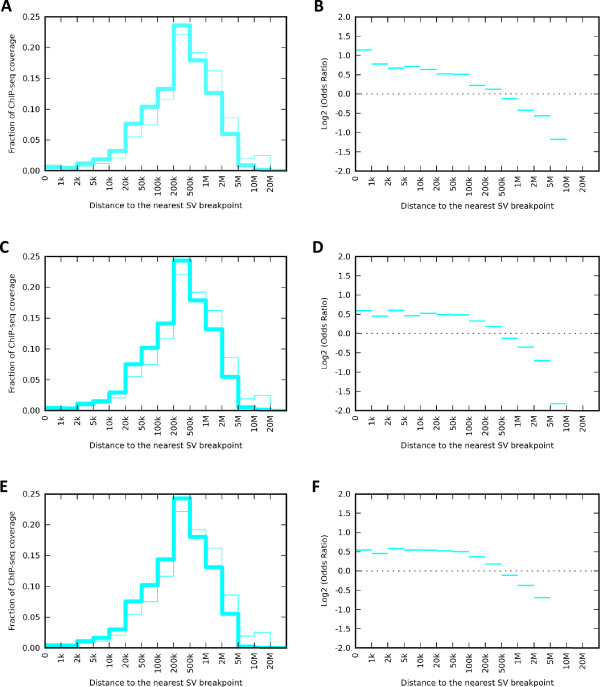
**Enrichment metrics as a function of distance in the Breast-Stephens SM callset.** Two enrichment metrics: fraction of coverage (left) and odds ratio (right) as a function of distance. Each bin on the horizontal axis is defined as a “range”, e.g. a marker between 100 kb and 200 kb depicts enrichment in regions >100 kb but <200 kb from the nearest SM breakpoint. **A**. Observed fraction of ChIP-seq (Pol II, GM12878, SYDH) coverage falling within given distance range of SM breakpoints (thick line) vs. the fraction expected to fall based on the total size of the genome in each bin. **B**. Enrichment odds ratio for ChIP-seq peaks (Pol II, GM12878, SYDH). **C**. Observed fraction of DNaseI peaks (HeLaS3, Duke) falling within given distance of SM breakpoints (thick line) vs. the fraction expected to fall based on the total size of the genome in each bin. **D**. Enrichment odds ratio for DNaseI peaks (HeLaS3, Duke). **E**. Observed fraction of FAIRE peaks (HeLaS3, UNC) falling within given distance of SM breakpoints (thick line) vs. the fraction expected to fall based on the total size of the genome in each bin. **F**. Enrichment odds ratio for FAIRE peaks (HeLaS3, UNC).

**Figure 8:**
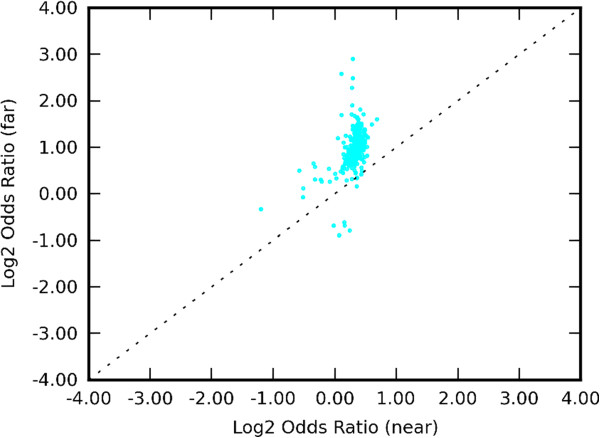
**Patterns of ChIP-seq enrichment extend up to 200 kb.** Odds ratio values across all available ChIP-seq experiments for the Breast-Stephens callset. Each point represents a different ChIP-seq experiment, with odds ratio calculated separately near (≤10 kb) genes (horizontal axis) and far from (>10 kb) genes (vertical axis). Positive values indicate enrichment of ChIP-seq signal within 200 kb of SM breakpoints.

Figure 7A and B demonstrates that ChIP-seq signal is enriched in the vicinity of SM breakpoints up to 200 kb in the Breast-Stephens callset. Also both DNase and FAIRE signal was enriched up to 200 kb (panels C-F). Similar enrichment patterns were observed in other genophilic cancers (data not shown). Furthermore, we calculated the enrichment odds ratio for all ChIP-seq experiments similarly to that shown in Figure 3 using alternate range cut-offs, namely 200 kb for distance from the breakpoints and 10 kb for distance from genes. The results are shown Figure 8, indicating that the pattern of enrichment odds ratio being higher far from genes than near genes holds at these longer distances as well. Similar results are found for all other SM callsets (Additional file 5).

## Discussion

We have performed computational experiments that have demonstrated enrichment of protein binding to DNA and open chromatin in the vicinity of SM breakpoints. More importantly, we have shown that protein binding and open chromatin enrichment in the vicinity of SM breakpoints is stronger far from genes than near genes, as exemplified in Figure 2A and C, Figure 3 and Figure 8. Overall, all three types of assays (protein binding ChIP-seq, DnaseI and FAIRE) showed similar patterns of locational enrichment with respect to each SM callset, although the dispersion of DnaseI was less than that of FAIRE or ChIP-seq. These results indicate protein binding events and open chromatin state as two common and widespread features that strongly correlate with SM formation across divergent cancer types.

To put our findings into perspective, we note that SM breakpoints have previously been shown to cluster in gene regions in the majority of cancer types [5]. However, our results are distinct from this effect. We studied two subtypes of prostate cancer with different molecular mechanisms of SM generation (chromoplexy and chromothripsis), one with enrichment of breakpoints in the gene regions and the other with depletion of breakpoints in the gene regions (Figure 2B and D). In both of these subtypes, we found protein binding sites to be more strongly localized to breakpoints in the regions far from the genes than in the regions near genes. Such behavior is robust across all cancer SM callsets, including those with lower baseline levels of protein-binding in the vicinity of breakpoints, such as the ETSwt/CHD1del subset of Prostate-Baca and also Head&Neck-Stransky. Therefore, our work reveals a genomic behavior that unifies divergent cancer types.

Although the effects of protein binding and open chromatin on breakpoint locations are entangled, since transcription factors are well known to typically bind in open chromatin, we have generalized the understanding of each feature. From the protein-binding perspective, previous studies have shown that androgen receptor binding promotes SM breakpoints [20]. Our results indicate that this phenomenon is not limited to the androgen receptor but is common to multiple proteins with diverse functions, including transcription (eg. Pol II), DNA repair (BRCA1) or 3D genome structure (CTCF) (Additional file 6). From the open chromatin perspective, previous work utilizing the I-SceI system in B cells [10, 13, 19] showed that breakpoints induced by addition of a sequence motif preferentially occurred in regions of actively transcribed chromatin. Our work shows that this active chromatin preference occurs in many cancers and is not specific to the details of the I-SceI system. Furthermore we found that sites with more proteins binding have a stronger effect size (Figure 4). Our results also raise an interesting point regarding the role of cell type. Although transcription factor binding is remarkably cell-type specific [33], recent studies have shown that 3D genome structure is less dynamic than protein binding [34]. We observe similar effect sizes when comparing behaviors of different proteins or different cell types (Additional file 6), suggesting the relationship between protein binding and SM formation is also mediated by a less dynamic variable such as 3D structure.

The locational tendencies of SM breakpoints in cancer are the product of both mutational and selective forces. We speculate that protein binding and open chromatin drive breakpoints at the mutational level. Breakpoints would then be subject to purifying selection within tumors, with a greater chance of being deleterious if they disrupt essential genes. This selection pressure may vary depending on cancer type, yielding fewer breakpoints in gene regions for cancers with greater sensitivity to gene disruption, i.e. the genophobic cancers, and more breakpoints in gene regions for cancers with lower sensitivity, i.e. the genophilic cancers. Such a mechanism would be consistent with the stronger effects far from genes. It would also explain why in the genophilic cancers breakpoints are generally closer to genes. This is because a large amount of protein binding is localized near gene regions at promoters, which could create the genophilic behavior at the mutational level. An ultimate future experiment to study this mutational effect directly would involve inserting known protein binding motifs into a cellular genome and arresting the cell cycle followed by single-cell sequencing to detect newly formed breakpoints. Design of such sequencing experiment remains challenging as even the most common DSBs occur in only 1 per 10,000 cells [10].

### Limitations

Our present study has certain important limitations. First, the open chromatin and protein ChIP-seq experiments were performed in a variety of cell lines, different from the cancers studied. In an attempt to understand the effect of cell line selection, we divided the cell lines into three categories: stem cells, lymphoblastoid (EBV-transformed) and cancer cell lines. This comparison (Additional file 7) shows that the log odds ratio difference (**Δ**) between the regions near and far from genes tends to be lower in the stem cell lines as compared to the lymphoblastoid and cancer cell lines.

The second limitation comes from the variety of sequencing platforms and algorithms used to identify SMs. Overall, three different pipelines were used: (a) the Broad Institute pipeline, used to generate Colorectal-Bass, HeadNeck-Stransky, Prostate-Berger and ProstateBaca, (b) the Wellcome Trust Sanger Institute pipeline used to generate Breast-Stephens, Breast-NikZainal and Ovarian-McBride and (c) the SOLiD-based pipeline used to generate Breast-Inaki. On the one hand, the reproducibility of the enrichment difference (near vs. far from genes) across at least three different platforms shows that our findings are not resulting from any pipeline-specific biases. On the other hand, interpretation of the differences between different pipelines must be approached with caution. The Wellcome Trust Sanger Institute pipeline has changed slightly over time (see read length and mapping in Table 1), while the Broad Institute pipeline (Colorectal-Bass, Prostate-Berger, Prostate-Baca and HeadNeck-Stransky) have been more stable. It is worth noting that within the Broad datasets we see distinct behaviors (Figure 2A and Figure 2C), indicating that pipeline choice does not drive the observations.

We also directly assessed the effect of SM caller on the observed enrichment by comparing the SM calls made using two SM calling algorithms (Hydra [35] and Meerkat [36]) in two cancers studied in The Cancer Genome Atlas, namely breast invasive carcinoma (“BRCA”) and Lung Squamous Cell Carcinoma (“LUSC”). The results shown in Additional file 8 again demonstrate the our findings are not sensitive to the choice of SM caller.

Conceptually, the robustness of our results across callers is likely because we are considering effects at a broader length scale (50 kb) than the typical scale of insert sizes (Table 1) in the paired end sequencing process. As a result, insert-related caller-specific uncertainties in the locations of SV breakpoints are likely averaged out in our analysis procedure. It is also important to note that the callsets used in our study may differ in the number of the SM events of various types and intrachromosomal lengths, our findings hold true if the interchromosomal events were considered alone, indicating that our results are not biased by the event length spectrum.

## Conclusions

Protein binding and open chromatin state are commonly associated with propensity for SM breakpoints. These effects appear to be common across cancers and not limited to androgen receptor binding or the I-SceI system, where they were originally described. Furthermore, the effect of functional chromatin state is robust over a wide range of distances around the SM breakpoints, extending up to 200 kb.

### Availability of supporting data

DNA-PET sequencing data of MB231 and MB436 are available in the NCBI Sequence Read Archive repository (SRA; http://www.ncbi.nlm.nih.gov/sra) under accession number PRJNA234462.

## Electronic supplementary material

Additional file 1: **Cumulative histogram of SM distances from breakpoints in the autosomal intrachromosomal SM callsets.** Cumulative histogram of SM distances from genes in the autosomal intrachromosomal SM callsets (“True SM”, blue line) vs. randomized controls (“CTRL”, dotted green line). Distances are with respect to the nearest gene. Results are shown for all SM callsets: Breast-Inaki (A), Breast-Stephens (B), Breast-NikZainal (C), Ovarian-McBride (D), Colorectal-Bass (E), Head&Neck-Stransky (F), Prostate-Berger (G), Prostate-Baca (H), ETSwt/CHD1del (I), and ETS+/CHD1wt (J). (PDF 1 MB)

Additional file 2: **Enrichment odds ratio for protein ChIP-seq and two open chromatin assays (DnaseI and FAIRE) in the vicinity of SMs in various callsets, separately for inter- and intrachromosomal events.** Enrichment of protein ChIP-seq and two open chromatin assays (DNaseI and FAIRE) signal in all SM callsets. Data in each cell show log_2_ odds ratio (mean ± standard deviation; positive values indicate enrichment). In each table row, only those protein binding ChIP-seq and open chromatin experiments, that have a non-zero entry in each cell of the 2 × 2 × 2 contingency table, were used; the number of such experiments is shown as *n*. Δ indicates difference of log OR between the regions near and far from genes. ^a^p-value calculated against a null hypothesis of log OR being 0 near genes. ^b^p-value calculated against a null hypothesis of log OR being 0 far from genes. ^c^ p-value calculated against a null hypothesis of no difference in odds ratio between near and far from genes. (PDF 64 KB)

Additional file 3: **Ranking of protein binding enrichment separated by SM callset.** The log2 odds ratio has been averaged over available ChIP-seq experiments, if more than one has been performed. In each SM callset, the top 10 proteins are highlighed green and the bottom 10 are highlighted red. (PDF 64 KB)

Additional file 4: **Histogram of chromatin state at the breakpoints in three different SM callsets.** Teal and white bars indicate observed vs. expected values, respectively. Erros bars indicate binomial standard error. Left panels show the full histograms, the right panels show respective zoom-in views at low frequency. (PDF 1 MB)

Additional file 5: **Patterns of ChIP-seq enrichment extend up to 200 kb.** Odds ratio values across all available protein binding ChIP-seq experiments. Each point represents a different protein ChIP-seq experiment, with odds ratio calculated separately near (≤10 kb) genes (horizontal axis) and far from (>10 kb) genes (vertical axis). Positive values indicate enrichment of protein ChIP-seq signal within 200 kb of SM breakpoints. Data shown in various SM callsets: Breast-Inaki (A), Breast-Stephens (B), Breast-NikZainal (C), Ovarian-McBride (D), Colorectal-Bass (E), Head&Neck-Stransky (F), Prostate-Berger (G), Prostate-Baca (H). (PDF 351 KB)

Additional file 6: **Enrichment of protein binding events in the vicinity of breakpoints is common across proteins with diverse functions, such as transcription (eg. Pol II), DNA repair (BRCA1) and 3D structure (CTCF).** Bars indicates fraction of ChIP-seq signal falling within 50 kb of any breakpoint. The horizontal line indicates baseline expectations, i.e. the fraction of the genome falling within that distance of any breakpoint. (PDF 205 KB)

Additional file 7: **Effect of cell type on enrichment of protein binding ChIP-seq signal.** Each point represents a different protein binding ChIP-seq experiment, with odds ratio calculated separately near genes (horizontal axis) and far from genes (vertical axis). Positive values indicate enrichment of protein ChIP-seq signal within 50 kb of SM breakpoints. Experiments performed in the stem cell lines are shown in the top row, in the cancer cell lines in the middle and in the EBV-transformed lymphoblastoid cell lines in the bottom row. Δ indicates the difference of log OR between the regions near and far from genes, i.e. the average location of the cloud of points above the diagonal line, averaged over *n* experiments. Data shown in various SM callsets: Breast-Inaki (A), Breast-Stephens (B), Breast-NikZainal (C), Ovarian-McBride (D), Colorectal-Bass (E), Head&Neck-Stransky (F), Prostate-Berger (G), Prostate-Baca (H). (PDF 392 KB)

Additional file 8: **Effect of SM calling pipeline.** Odds ratio values across all available protein binding ChIP-seq experiments. Each point represents a different protein binding ChIP-seq experiment, with odds ratio calculated separately near (≤60 kb) genes (horizontal axis) and far from (>60 kb) genes (vertical axis). Positive values indicate enrichment of protein ChIP-seq signal within 50 kb of SM breakpoints. Data shown in two cancers from The Cancer Genome Atlas (breast cancer “BRCA” in the top row and lung cancer “LUSC” in the bottom row) using two different SM callers (Hydra on the left and Meerkat on the right). (PDF 97 KB)
