# Supplementary material for: Functional chromatin features are associated with structural mutations in cancer
Source: BMC Genomics. 2014 Nov 23;15(1):1013. doi: 10.1186/1471-2164-15-1013 (PMC4253614; doi:10.1186/1471-2164-15-1013)
Supplement: Supplementary file 1 — Additional file 1: Cumulative histogram of SM distances from breakpoints in the autosomal intrachromosomal SM callsets. Cumulative histogram of SM distances from genes in the autosomal intrachromosomal SM callsets (“True SM”, blue line) vs. randomized controls (“CTRL”, dotted green line). Distances are with respect to the nearest gene. Results are shown for all SM callsets: Breast-Inaki (A), Breast-Stephens (B), Breast-NikZainal (C), Ovarian-McBride (D), Colorectal-Bass (E), Head&Neck-Stransky (F), Prostate-Berger (G), Prostate-Baca (H), ETSwt/CHD1del (I), and ETS+/CHD1wt (J). (PDF 1 MB) [file 12864_2014_6709_MOESM1_ESM.pdf]

Additional File 1

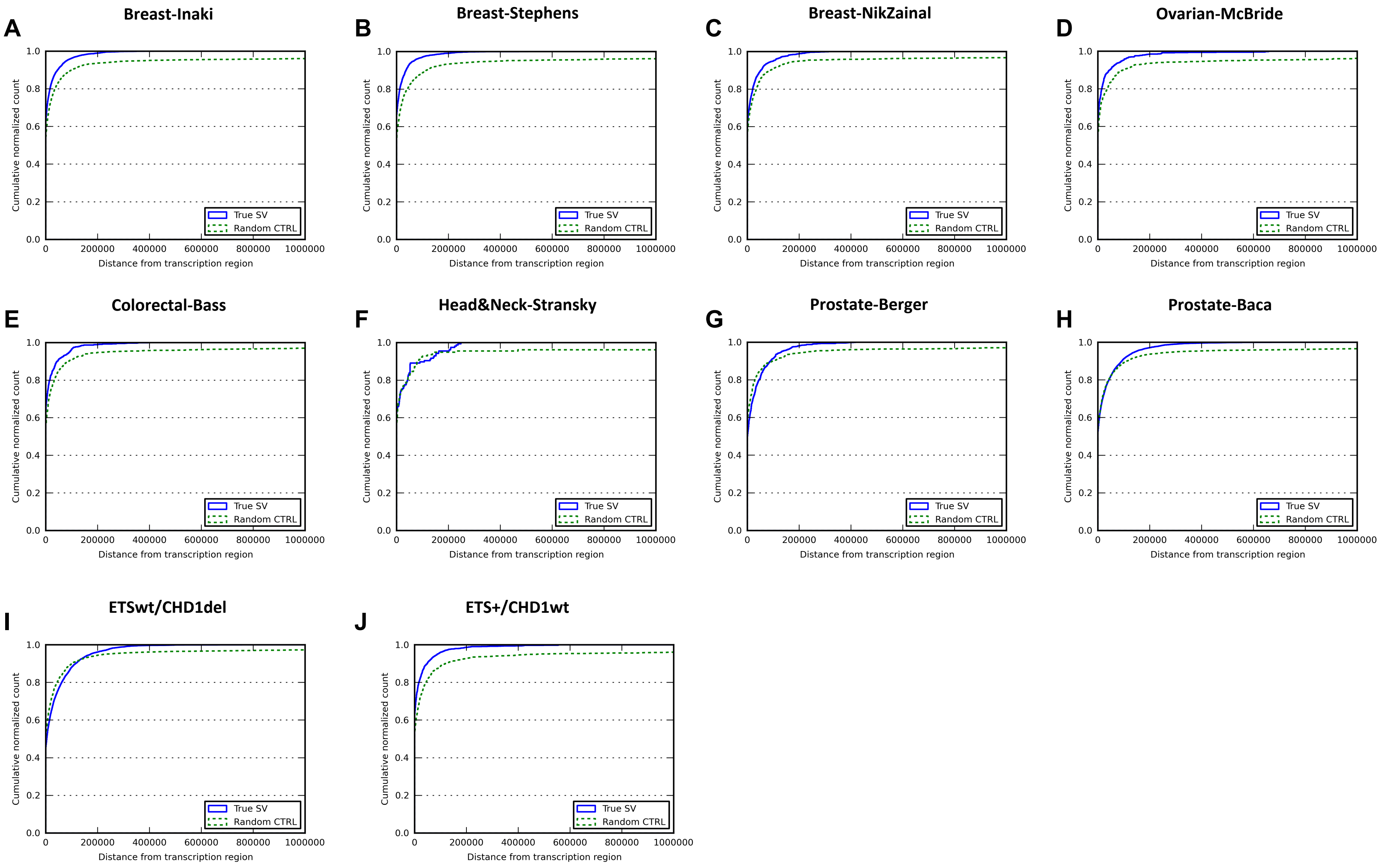

Cumulative histogram of SM distances from genes in the autosomal intrachromosomal SM callsets ("True SM", blue line). Randomized controls are also shown ("CTRL", dotted green line). Distances are with respect to the nearest gene. Results are shown for all SM callsets: Breast-Inaki (A), Breast-Stephens (B), Breast-NikZainal (C), Ovarian-McBride (D), Colorectal-Bass (E), Head&Neck-Stransky (F), Prostate-Berger (G), Prostate-Baca (H), ETSwt/CHD1del (I), and ETS+/CHD1wt (J).
