# Supplementary material for: Functional chromatin features are associated with structural mutations in cancer
Source: BMC Genomics. 2014 Nov 23;15(1):1013. doi: 10.1186/1471-2164-15-1013 (PMC4253614; doi:10.1186/1471-2164-15-1013)
Supplement: Supplementary file 2 — Additional file 2: Enrichment odds ratio for protein ChIP-seq and two open chromatin assays (DnaseI and FAIRE) in the vicinity of SMs in various callsets, separately for inter- and intrachromosomal events. Enrichment of protein ChIP-seq and two open chromatin assays (DNaseI and FAIRE) signal in all SM callsets. Data in each cell show log2 odds ratio (mean ± standard deviation; positive values indicate enrichment). In each table row, only those protein binding ChIP-seq and open chromatin experiments, that have a non-zero entry in each cell of the 2 × 2 × 2 contingency table, were used; the number of such experiments is shown as n. Δ indicates difference of log OR between the regions near and far from genes. ap-value calculated against a null hypothesis of log OR being 0 near genes. bp-value calculated against a null hypothesis of log OR being 0 far from genes. c p-value calculated against a null hypothesis of no difference in odds ratio between near and far from genes. (PDF 64 KB) [file 12864_2014_6709_MOESM2_ESM.pdf]

Additional files

Additional file 1 – Cumulative histogram of SM distances from breakpoints in the autosomal intrachromosomal SM callsets

Cumulative histogram of SM distances from genes in the autosomal intrachromosomal SM callsets (“True SM”, blue line) vs. randomized controls (“CTRL”, dotted green line). Distances are with respect to the nearest gene. Results are shown for all SM callsets: Breast-Inaki (A), Breast-Stephens (B), Breast-NikZainal (C), Ovarian-McBride (D), Colorectal-Bass (E), Head&Neck-Stransky (F), Prostate-Berger (G), Prostate-Baca (H), ETSwt/CHD1del (I), and ETS+/CHD1wt (J).

Additional file 2 – Enrichment odds ratio for protein ChIP-seq and two open chromatin assays (DnaseI and FAIRE) in the vicinity of SMs in various callsets, separately for inter- and intrachromosomal events.

Enrichment of protein ChIP-seq and two open chromatin assays (DNaseI and FAIRE) signal in all SM callsets. Data in each cell show $\log_2$ odds ratio (mean ± standard deviation; positive values indicate enrichment). In each table row, only those protein binding ChIP-seq and open chromatin experiments, that have a non-zero entry in each cell of the 2×2×2 contingency table, were used; the number of such experiments is shown as *n*. **Δ** indicates difference of log OR between the regions near and far from genes.

[a] p-value calculated against a null hypothesis of log OR being 0 near genes.

[b] p-value calculated against a null hypothesis of log OR being 0 far from genes.

[c] p-value calculated against a null hypothesis of no difference in odds ratio between near and far from genes.

	ChIP-seq log2 OR			DnaseI log2 OR			Faire log2 OR		
**SM callset**	**Near[a]**	**Far[b]**	**Δ[c] (far-near)**	**Near[a]**	**Far[b]**	**Δ[c] (far-near)**	**Near[a]**	**Far[b]**	**Δ[c] (far-near)**
interchromosomal events									
Breast-Inaki	0.30 ± 0.20<br>p=6.9E-86<br>n=323	0.59 ± 0.97<br>p=1.3E-23<br>n=323	0.29 ± 0.96<br>p=1.9E-07<br>n=323	0.24 ± 0.10<br>p=8.6E-52<br>n=122	0.63 ± 0.24<br>p=3.3E-57<br>n=122	0.40 ± 0.19<br>p=9.8E-47<br>n=122	0.20 ± 0.32<br>p=1.2E-02<br>n=19	0.53 ± 0.39<br>p=1.6E-05<br>n=19	0.32 ± 0.24<br>p=1.7E-05,<br>n=19
Colorectal-Bass	0.01 ± 0.26<br>p=5.4E-01<br>n=211	0.98 ± 1.20<br>p=2.9E-25<br>n=211	0.97 ± 1.23<br>p=4.7E-24<br>n=211	-0.01 ± 0.07<br>p=3.5E-01<br>n=122	0.92 ± 0.35<br>p=1.6E-56,<br>n=122	0.92 ± 0.35<br>p=3.2E-56<br>n=122	-0.07 ± 0.31<br>p=3.5E-01<br>n=19	0.68 ± 0.75<br>p=9.1E-04<br>n=19	0.75 ± 0.86<br>p=1.3E-03<br>n=19
HeadNeck-Stransky	-0.33 ± 0.28<br>p=1.2E-20<br>n=101	0.40 ± 0.87<br>p=1.3E-05<br>n=101	0.73 ± 0.90<br>p=9.4E-13<br>n=101	-0.22 ± 0.10<br>p=9.7E-48<br>n=120	-0.20 ± 0.74<br>p=3.5E-03<br>n=120	0.02 ± 0.73<br>p=7.5E-01<br>n=120	-0.18 ± 0.40<br>p=6.6E-02<br>n=19	-0.10 ± 1.06<br>p=6.7E-01<br>n=19	0.07 ± 0.86<br>p=7.1E-01<br>n=19
ETSwt/CHD 1del subset of Prostate-Baca	-0.61 ± 0.28<br>p=4.0E-100<br>n=261	0.14 ± 0.96<br>p=2.1E-02<br>n=261	0.74 ± 1.00<br>p=6.0E-27<br>n=261	-0.58 ± 0.11<br>p=2.6E-92<br>n=122	0.02 ± 0.36<br>p=5.6E-01<br>n=122	0.60 ± 0.38<br>p=7.5E-35<br>n=122	-0.33 ± 0.37<br>p=1.0E-03<br>n=19	-0.14 ± 0.60<br>p=3.2E-01<br>n=19	0.19 ± 0.45<br>p=7.3E-02<br>n=19
ETS+/CHD1 wt subset of Prostate-Baca	0.40 ± 0.17<br>p=1.5E-123<br>n=305	1.11 ± 0.90<br>p=2.8E-63<br>n=305	0.71 ± 0.89<br>p=1.1E-34<br>n=305	0.30 ± 0.06<br>p=4.5E-91<br>n=122	0.92 ± 0.23<br>p=1.7E-75<br>n=122	0.62 ± 0.24<br>p=7.3E-56<br>n=122	0.25 ± 0.13<br>p=8.2E-08<br>n=19	0.98 ± 0.48<br>p=4.7E-08<br>n=19	0.73 ± 0.44<br>p=8.8E-07<br>n=19
Ovarian-McBride	0.45 ± 0.26<br>p=1.5E-65<br>n=210	1.12 ± 1.11<br>p=8.0E-34<br>n=210	0.67 ± 1.05<br>p=2.0E-17<br>n=210	0.36 ± 0.19<br>p=3.3E-42<br>n=122	0.66 ± 0.35<br>p=2.6E-41<br>n=122	0.30 ± 0.30<br>p=1.1E-19<br>n=122	0.21 ± 0.32<br>p=1.1E-02<br>n=19	0.58 ± 0.58<br>p=3.6E-04<br>n=19	0.37 ± 0.72<br>p=3.8E-02<br>n=19
Breast-NikZainal	0.21 ± 0.18<br>p=3.8E-54<br>n=284	1.23 ± 0.84<br>p=4.4E-73<br>n=284	1.02 ± 0.83<br>p=9.9E-59<br>n=284	0.18 ± 0.05<br>p=1.5E-66<br>n=122	1.09 ± 0.27<br>p=1.7E-77<br>n=122	0.91 ± 0.25<br>p=1.2E-72<br>n=122	0.12 ± 0.26<br>p=5.6E-02<br>n=19	1.06 ± 0.59<br>p=3.5E-07<br>n=19	0.93 ± 0.40<br>p=6.0E-09<br>n=19
Prostate-Berger	0.13 ± 0.22<br>p=9.2E-18<br>n=247	1.53 ± 1.07<br>p=1.3E-61<br>n=247	1.40 ± 1.05<br>p=6.0E-57<br>n=247	0.13 ± 0.09<br>p=2.8E-31<br>n=122	0.82 ± 0.40<br>p=1.6E-45<br>n=122	0.70 ± 0.39<br>p=4.6E-39<br>n=122	-0.02 ± 0.33<br>p=8.4E-01<br>n=19	0.65 ± 0.91<br>p=6.4E-03<br>n=19	0.66 ± 0.86<br>p=3.6E-03<br>n=19
Prostate-Baca	0.30 ± 0.17<br>p=9.5E-111<br>n=347	0.59 ± 0.78<br>p=5.8E-36<br>n=347	0.29 ± 0.77<br>p=1.1E-11<br>n=347	0.17 ± 0.05<br>p=9.9E-72<br>n=122	0.57 ± 0.15<br>p=6.7E-74<br>n=122	0.39 ± 0.15<br>p=2.3E-56<br>n=122	0.16 ± 0.10<br>p=1.6E-06<br>n=19	0.58 ± 0.48<br>p=6.4E-05<br>n=19	0.42 ± 0.43<br>p=5.8E-04<br>n=19
Breast-Stephens	0.33 ± 0.14<br>p=5.5E-138<br>n=339	1.54 ± 0.93<br>p=4.0E-99<br>n=339	1.21 ± 0.93<br>p=8.4E-75<br>n=339	0.30 ± 0.07<br>p=3.3E-82<br>n=122	1.14 ± 0.26<br>p=5.6E-82<br>n=122	0.85 ± 0.24<br>p=4.2E-71<br>n=122	0.25 ± 0.31<br>p=2.2E-03<br>n=19	1.37 ± 0.61<br>p=1.3E-08<br>n=19	1.12 ± 0.40<br>p=4.0E-10<br>n=19

	ChIP-seq log_2 OR			DnaseI log_2 OR			Faire log_2 OR		
**SM callset**	**Near[a]**	**Far[b]**	**Δ[c] (far-near)**	**Near[a]**	**Far[b]**	**Δ[c] (far-near)**	**Near[a]**	**Far[b]**	**Δ[c] (far-near)**
intrachromosomal events									
Breast-Inaki	0.37 ± 0.14 p=7.9E-171 n=385	1.00 ± 0.73 p=1.9E-90 n=385	0.64 ± 0.71 p=8.8E-52 n=385	0.28 ± 0.06 p=3.6E-88 n=122	0.90 ± 0.14 p=2.1E-101 n=122	0.62 ± 0.15 p=1.5E-79 n=122	0.24 ± 0.21 p=1.0E-04 n=19	0.94 ± 0.43 p=1.6E-08 n=19	0.70 ± 0.30 p=8.7E-09 n=19
Colorectal-Bass	0.12 ± 0.17 p=1.4E-31 n=337	1.18 ± 0.98 p=5.1E-67 n=337	1.06 ± 0.99 p=2.1E-57 n=337	0.02 ± 0.06 p=4.4E-05 n=122	0.99 ± 0.24 p=2.2E-77 n=122	0.97 ± 0.26 p=7.8E-74 n=122	0.02 ± 0.33 p=8.1E-01 n=19	1.22 ± 0.47 p=1.3E-09 n=19	1.20 ± 0.49 p=3.1E-09 n=19
HeadNeck-Stransky	-0.44 ± 0.40 p=8.1E-39 n=212	1.57 ± 1.55 p=1.6E-34 n=212	2.02 ± 1.71 p=8.5E-42 n=212	-0.31 ± 0.14 p=5.3E-49 n=122	0.32 ± 0.81 p=3.3E-05 n=122	0.63 ± 0.79 p=1.8E-14 n=122	-0.33 ± 0.29 p=1.0E-04 n=19	0.20 ± 0.91 p=3.5E-01 n=19	0.52 ± 0.84 p=1.4E-02 n=19
ETSwt/CHD1del subset of Prostate-Baca	-0.90 ± 0.34 p=5.4E-181 n=400	0.08 ± 0.71 p=3.4E-02 n=400	0.98 ± 0.66 p=1.5E-103 n=400	-0.73 ± 0.23 p=7.3E-65 n=122	0.17 ± 0.30 p=1.4E-08 n=122	0.90 ± 0.16 p=8.4E-93 n=122	-0.41 ± 0.47 p=1.4E-03 n=19	0.41 ± 0.44 p=6.8E-04 n=19	0.83 ± 0.26 p=6.0E-11 n=19
ETS+/CHD1wt subset of Prostate-Baca	0.38 ± 0.15 p=2.7E-144 n=318	1.01 ± 0.79 p=1.0E-68 n=318	0.63 ± 0.79 p=9.7E-36 n=318	0.29 ± 0.05 p=3.3E-94 n=122	0.96 ± 0.18 p=6.0E-91 n=122	0.67 ± 0.16 p=1.5E-78 n=122	0.19 ± 0.19 p=3.3E-04 n=19	0.85 ± 0.42 p=6.6E-08 n=19	0.65 ± 0.30 p=1.5E-08 n=19
Ovarian-McBride	0.38 ± 0.15 p=2.5E-146 n=344	1.44 ± 0.87 p=7.2E-101 n=344	1.07 ± 0.85 p=1.2E-72 n=344	0.28 ± 0.05 p=5.4E-93 n=122	1.12 ± 0.33 p=5.6E-69 n=122	0.84 ± 0.30 p=7.9E-59 n=122	0.18 ± 0.23 p=4.1E-03 n=19	1.02 ± 0.40 p=1.5E-09 n=19	0.85 ± 0.23 p=3.9E-12 n=19
Breast-NikZainal	0.13 ± 0.14 p=7.8E-50 n=357	1.30 ± 1.00 p=1.2E-78 n=357	1.17 ± 1.02 p=1.5E-66 n=357	0.13 ± 0.08 p=1.7E-39 n=122	1.12 ± 0.18 p=1.0E-97 n=122	0.99 ± 0.21 p=1.2E-85 n=122	0.10 ± 0.16 p=1.7E-02 n=19	1.12 ± 0.52 p=2.1E-08 n=19	1.02 ± 0.44 p=7.6E-09 n=19
Prostate-Berger	-0.36 ± 0.24 p=3.5E-83 n=320	0.31 ± 0.88 p=6.9E-10 n=320	0.67 ± 0.89 p=2.5E-33 n=320	-0.34 ± 0.19 p=6.2E-39 n=122	0.29 ± 0.31 p=1.6E-18 n=122	0.63 ± 0.22 p=2.9E-60 n=122	-0.14 ± 0.41 p=1.6E-01 n=19	0.44 ± 0.51 p=1.5E-03 n=19	0.58 ± 0.47 p=4.4E-05 n=19
Prostate-Baca	-0.24 ± 0.14 p=2.7E-122 n=411	0.34 ± 0.62 p=3.7E-25 n=411	0.58 ± 0.59 p=4.1E-62 n=411	-0.25 ± 0.12 p=1.8E-46 n=122	0.40 ± 0.27 p=2.8E-32 n=122	0.64 ± 0.18 p=1.6E-71 n=122	-0.13 ± 0.28 p=6.3E-02 n=19	0.54 ± 0.39 p=8.7E-06 n=19	0.67 ± 0.22 p=6.7E-11 n=19
Breast-Stephens	0.38 ± 0.12 p=3.1E-195 n=368	1.14 ± 0.77 p=2.6E-95 n=368	0.76 ± 0.77 p=5.8E-56 n=368	0.33 ± 0.04 p=5.3E-117 n=122	1.20 ± 0.15 p=8.1E-113 n=122	0.88 ± 0.13 p=5.1E-102 n=122	0.27 ± 0.20 p=1.2E-05 n=19	1.14 ± 0.23 p=2.6E-14 n=19	0.87 ± 0.16 p=4.0E-15 n=19
