# Supplementary material for: Functional chromatin features are associated with structural mutations in cancer
Source: BMC Genomics. 2014 Nov 23;15(1):1013. doi: 10.1186/1471-2164-15-1013 (PMC4253614; doi:10.1186/1471-2164-15-1013)
Supplement: Supplementary file 3 — Additional file 3: Ranking of protein binding enrichment separated by SM callset. The log2 odds ratio has been averaged over available ChIP-seq experiments, if more than one has been performed. In each SM callset, the top 10 proteins are highlighed green and the bottom 10 are highlighted red. (PDF 64 KB) [file 12864_2014_6709_MOESM3_ESM.pdf]

Ranking of protein binding enrichment separated by SM callset.

The log2 odds ratio has been averaged over available ChIP-seq experiments, if more than one has been performed.

In each SM callset, the top 10 proteins are highlighed green and the bottom 10 are highlighted red.

Breast-Inaki		Breast-Stephens		Breast-NikZainal		Ovarian-McBride		Colorectal-Bass		HeadNeck-Stransky		Prostate-Berger		Prostate-Baca	
ChIP-seq protein	Log2 OR	ChIP-seq protein	Log2 OR	ChIP-seq protein	Log2 OR	ChIP-seq protein	Log2 OR	ChIP-seq protein	Log2 OR	ChIP-seq protein	Log2 OR	ChIP-seq protein	Log2 OR	ChIP-seq protein	Log2 OR
ATF3	0.557	ATF3	0.629	ATF3	0.279	ATF3	0.627	ATF3	0.279	BATF	-0.308	BATF	-0.133	ATF3	-0.099
BATF	0.323	BATF	0.459	BATF	0.327	BATF	0.270	BATF	0.122	BCL11A	0.327	BCL11A	-0.103	BATF	-0.015
BCL11A	0.319	BCL11A	0.461	BCL11A	0.399	BCL11A	0.277	BCL11A	0.273	CCNT2	-0.285	BCL3	-0.274	BCL11A	0.091
BCL3	0.654	BCL3	0.654	BCL3	0.311	BCL3	0.577	BCL3	0.441	CEBPB	-0.273	BHLHE40	-0.560	BCL3	-0.063
BCLAF1	0.617	BCLAF1	0.624	BCLAF1	0.226	BCLAF1	0.632	BCLAF1	0.406	CHD2	0.170	BRCA1	-0.306	BCLAF1	0.046
BHLHE40	0.552	BHLHE40	0.752	BHLHE40	0.440	BHLHE40	0.754	BRCA1	0.083	POL3	-1.186	BRF2	0.523	BHLHE40	-0.308
BRCA1	0.599	BRCA1	0.598	BRCA1	0.414	BRCA1	0.576	CCNT2	0.099	CTCF	-0.182	CCNT2	-0.276	BRCA1	0.009
BRF2	-0.005	CCNT2	0.608	CCNT2	0.349	CCNT2	0.609	CEBPB	0.207	E2F6	-0.480	CEBPB	-0.141	BRF2	-0.072
CCNT2	0.525	CEBPB	0.513	CEBPB	0.368	CEBPB	0.401	CHD2	0.318	EBF	-0.162	CHD2	-0.302	CCNT2	-0.103
CEBPB	0.439	CHD2	0.602	CHD2	0.361	POL3	0.268	CTBP2	0.511	EBF1	-0.296	POL3	-0.240	CEBPB	-0.057
CHD2	0.588	POL3	0.509	POL3	0.301	CTBP2	0.575	CTCF	0.182	EFOS	-0.548	CTBP2	0.034	CHD2	-0.059
POL3	0.525	CTBP2	0.537	CTBP2	0.326	CTCF	0.468	E2F1	0.267	EGATA2	-0.266	CTCF	-0.160	POL3	-0.146
CTBP2	0.275	CTCF	0.451	CTCF	0.288	E2F1	0.513	E2F6	0.084	EGR1	-0.347	E2F6	-0.374	CTBP2	-0.057
CTCF	0.371	E2F1	0.721	E2F1	0.381	E2F6	0.618	EBF	0.180	EJUNB	-0.723	EBF	-0.340	CTCF	0.020
E2F4	0.618	E2F6	0.587	E2F6	0.249	EBF	0.461	EBF1	0.167	EJUND	-0.590	EBF1	-0.408	E2F1	-0.018
E2F6	0.585	EBF	0.570	EBF	0.268	EBF1	0.562	EFOS	0.126	ELF1	-0.412	EFOS	-0.156	E2F4	-0.046
EBF	0.460	EBF1	0.602	EBF1	0.289	EFOS	0.371	EGATA2	0.189	EP300	-0.079	EGATA2	-0.010	E2F6	-0.122
EBF1	0.498	EFOS	0.586	EFOS	0.281	EGATA2	0.521	EGR1	0.393	ESR1	-0.417	EGR1	-0.535	EBF	-0.084
EFOS	0.456	EGATA2	0.489	EGATA2	0.271	EGR1	0.759	EJUNB	0.114	FOS	-0.094	EJUNB	-0.169	EBF1	-0.102
EGATA2	0.312	EGR1	0.562	EGR1	0.239	EJUNB	0.482	EJUND	0.116	FOSL1	-0.911	EJUND	-0.127	EFOS	-0.264
EGR1	0.482	EHDAC8	0.232	EJUNB	0.335	EJUND	0.549	ELF1	0.256	FOXA1	-0.301	ELF1	-0.366	EGATA2	-0.253
EHDAC8	0.478	EJUNB	0.525	EJUND	0.419	ELF1	0.630	ELK4	-0.049	FOXA2	-0.395	ELK4	-0.031	EGR1	-0.110
EJUNB	0.410	EJUND	0.557	ELF1	0.314	ELK4	0.630	EP300	0.377	GABPA	-0.495	EP300	-0.131	EHDAC8	-0.035
EJUND	0.436	ELF1	0.617	ELK4	0.322	EP300	0.360	ESR1	-0.006	GATA1	-0.278	ESR1	-0.048	EJUNB	-0.189
ELF1	0.578	ELK4	0.621	EP300	0.396	ESR1	0.296	FOS	0.086	GATA2	-0.196	FAM48A	0.344	EJUND	-0.177
ELK4	0.683	EP300	0.499	ESR1	0.406	ESRRA	0.751	FOSL1	0.244	GATA3	-0.205	FOS	0.016	ELF1	-0.075
EP300	0.415	ESR1	0.610	ESRRA	0.199	FOS	0.466	FOSL2	0.374	GTF3C2	-0.531	FOSL1	-0.366	ELK4	0.050
ESR1	0.476	ESRRA	0.614	FAM48A	0.610	FOSL1	0.394	FOXA1	0.242	HDAC2	-0.558	FOSL2	-0.369	EP300	-0.040
ESRRA	0.751	ETS1	0.650	FOS	0.265	FOSL2	0.441	FOXA2	0.256	HEY1	-0.473	FOXA1	-0.069	ESR1	-0.088
FAM48A	0.246	FAM48A	-0.453	FOSL1	0.372	FOXA1	0.385	GABPA	0.280	HMGN3	-0.295	FOXA2	-0.122	ESRRA	-0.372
FOS	0.456	FOS	0.456	FOSL2	0.418	FOXA2	0.343	GATA1	0.185	HNF4A	-0.301	GABPA	-0.381	ETS1	0.012
FOSL1	0.496	FOSL1	0.621	FOXA1	0.409	GABPA	0.700	GATA2	0.072	IRF1	-0.068	GATA1	-0.282	FAM48A	0.855
FOSL2	0.516	FOSL2	0.567	FOXA2	0.442	GATA1	0.613	GATA3	0.138	IRF4	-0.463	GATA2	-0.095	FOS	-0.158
FOXA1	0.419	FOXA1	0.499	GABPA	0.201	GATA2	0.465	GTF2B	0.136	JUN	-0.274	GATA3	0.112	FOSL1	-0.242
FOXA2	0.388	FOXA2	0.487	GATA1	0.407	GATA3	0.433	GTF2F1	0.185	JUND	-0.174	GTF2F1	-0.351	FOSL2	-0.175
GABPA	0.567	GABPA	0.569	GATA2	0.368	GTF2F1	0.721	GTF3C2	0.658	KAP1	0.010	GTF3C2	-0.295	FOXA1	-0.034
GATA1	0.484	GATA1	0.639	GATA3	0.444	GTF3C2	0.368	HAE2F1	0.146	MAFF	0.053	HAE2F1	-0.223	FOXA2	-0.070
GATA2	0.297	GATA2	0.489	GTF2B	0.352	HAE2F1	0.608	HDAC2	0.370	MAFK	-0.087	HDAC2	-0.214	GABPA	-0.055
GATA3	0.453	GATA3	0.576	GTF2F1	0.415	HDAC2	0.542	HEY1	0.315	MAX	-0.248	HEY1	-0.269	GATA1	-0.119
GTF2B	0.512	GTF2B	0.716	GTF3C2	0.332	HEY1	0.598	HNF4A	0.274	MEF2A	-0.518	HNF4A	-0.403	GATA2	-0.132

GTF2F1	0.725	GTF2F1	0.719	HAE2F1	0.268	HMGN3	0.716	HNF4G	0.311	MEF2C	-0.335	HNF4G	-0.357	GATA3	0.015
GTF3C2	0.798	GTF3C2	0.449	HDAC2	0.409	HNF4A	0.458	IRF1	0.660	MXI1	-0.358	IRF1	-0.199	GTF2B	0.021
HAE2F1	0.612	HAE2F1	0.621	HEY1	0.286	HNF4G	0.454	IRF4	0.153	MYC	-0.484	IRF4	-0.212	GTF2F1	0.013
HDAC2	0.437	HDAC2	0.555	HMGN3	0.356	IRF1	0.694	JUN	0.151	NANOG	-0.399	JUN	-0.083	GTF3C2	0.086
HEY1	0.624	HEY1	0.616	HNF4A	0.413	IRF4	0.477	JUND	0.248	NFE2	-0.814	JUND	-0.224	HAE2F1	-0.083
HMGN3	0.584	HMGN3	0.607	HNF4G	0.439	JUN	0.315	KAP1	0.235	NFKB	-0.177	KAP1	-0.121	HDAC2	-0.072
HNF4A	0.488	HNF4A	0.633	IRF1	0.266	JUND	0.402	MAFF	0.270	NFYA	-0.086	MAFF	0.101	HEY1	-0.073
HNF4G	0.484	HNF4G	0.579	IRF4	0.230	KAP1	0.321	MAFK	0.166	NFYB	-0.242	MAFK	0.067	HMGN3	-0.106
HSF1	0.651	IRF1	0.620	JUN	0.295	MAFF	0.149	MAX	0.197	NR3C1	-0.458	MAX	-0.304	HNF4A	-0.182
IRF1	0.575	IRF4	0.523	JUND	0.306	MAFK	0.195	MEF2A	0.114	OCT2	-0.047	MEF2A	-0.153	HNF4G	-0.164
IRF3	0.795	JUN	0.536	KAP1	0.346	MAX	0.632	MEF2C	0.065	PAX5	-0.230	MEF2C	-0.183	IRF1	-0.132
IRF4	0.414	JUND	0.521	MAFF	0.279	MEF2A	0.425	MXI1	0.165	POL2	-0.618	MXI1	-0.347	IRF4	-0.007
JUN	0.427	KAP1	0.418	MAFK	0.297	MEF2C	0.406	MYC	0.219	POU2F2	-0.052	MYC	-0.332	JUN	-0.141
JUND	0.477	MAFF	0.248	MAX	0.330	MXI1	0.607	NANOG	0.382	POU5F1	0.037	NANOG	-0.176	JUND	-0.023
KAP1	0.285	MAFK	0.301	MEF2A	0.275	MYC	0.592	NFKB	0.239	RAD21	-0.130	NFKB	-0.300	KAP1	-0.032
MAFF	0.179	MAX	0.642	MEF2C	0.341	NANOG	0.405	NFYA	0.077	REST	-0.524	NFYA	-0.181	MAFF	0.145
MAFK	0.214	MEF2A	0.536	MXI1	0.399	NFE2	0.394	NFYB	0.195	RFX5	-0.273	NFYB	-0.320	MAFK	0.095
MAX	0.583	MEF2C	0.492	MYC	0.401	NFKB	0.572	NR3C1	0.152	SETDB1	-0.138	NR3C1	-0.212	MAX	-0.109
MEF2A	0.430	MXI1	0.733	NANOG	0.314	NFYA	0.632	OCT2	0.205	SIN3AK20	-0.126	NRF1	0.169	MEF2A	0.058
MEF2C	0.348	MYC	0.658	NFE2	0.344	NFYB	0.710	PAX5	0.276	SIRT6	0.194	OCT2	-0.139	MEF2C	0.076
MXI1	0.641	NANOG	0.401	NFKB	0.320	NR2C2	0.572	PBX3	0.287	SMC3	-0.217	PAX5	-0.309	MXI1	-0.067
MYC	0.648	NFE2	0.616	NFYA	0.179	NR3C1	0.338	POL2	0.236	SP1	-0.041	PBX3	-0.530	MYC	-0.082
NANOG	0.356	NFKB	0.557	NFYB	0.146	NRF1	0.605	POU2F2	0.198	SPI1	-0.258	POL2	-0.288	NANOG	0.023
NFE2	0.317	NFYA	0.424	NR2C2	0.480	OCT2	0.576	POU5F1	0.387	SRF	-0.221	POU2F2	-0.133	NFE2	-0.172
NFKB	0.474	NFYB	0.497	NR3C1	0.460	PAX5	0.509	PRDM1	0.218	STAT1	-0.284	POU5F1	0.126	NFKB	-0.017
NFYA	0.458	NR2C2	0.531	NRF1	0.390	PBX3	0.615	RAD21	0.156	STAT2	-0.083	PRDM1	0.114	NFYA	-0.069
NFYB	0.499	NR3C1	0.661	OCT2	0.284	POL2	0.702	REST	0.309	STAT3	-0.176	RAD21	-0.098	NFYB	-0.166
NR2C2	0.388	NRF1	0.639	PAX5	0.288	POU2F2	0.574	RFX5	0.276	TAF1	-0.368	REST	-0.546	NR2C2	-0.241
NR3C1	0.561	OCT2	0.605	PBX3	0.297	POU5F1	0.028	RPC155	0.736	TAF7	-0.569	RFX5	-0.122	NR3C1	-0.145
NRF1	0.627	PAX5	0.577	POL2	0.223	PRDM1	0.287	RXRA	0.372	TAL1	-0.322	RPC155	-0.339	NRF1	-0.008
OCT2	0.558	PBX3	0.609	POU2F2	0.281	RAD21	0.416	SETDB1	0.267	TBP	-0.535	RXRA	-0.368	OCT2	-0.013
PAX5	0.507	POL2	0.654	POU5F1	0.213	REST	0.617	SIN3AK20	0.350	TCF12	-0.083	SETDB1	-0.125	PAX5	-0.068
PBX3	0.547	POU2F2	0.607	PRDM1	0.602	RFX5	0.685	SIRT6	0.387	TCF7L2	-0.370	SIN3AK20	-0.413	PBX3	-0.117
PGC1A	0.594	POU5F1	0.182	RAD21	0.305	RPC155	0.374	SIX5	0.335	TFAP2C	-0.416	SIRT6	-0.208	PGC1A	-0.284
POL2	0.664	PRDM1	0.413	REST	0.153	RXRA	0.642	SMARCB1	0.068	USF1	-0.400	SIX5	-0.232	POL2	0.010
POU2F2	0.553	RAD21	0.447	RFX5	0.338	SETDB1	0.162	SMARCC1	0.082	USF2	-0.163	SMARCA4	0.017	POU2F2	-0.015
POU5F1	0.260	REST	0.434	RPC155	0.603	SIN3AK20	0.674	SMARCC2	0.066	YY1	-0.542	SMARCB1	-0.184	POU5F1	0.080
PRDM1	0.387	RFX5	0.708	RXRA	0.327	SIRT6	0.678	SMC3	0.180	ZBTB7A	-0.270	SMARCC1	-0.097	PRDM1	0.090
RAD21	0.348	RPC155	0.566	SETDB1	0.108	SIX5	0.826	SP1	0.331	ZEB1	-0.769	SMARCC2	-0.520	RAD21	0.029
REST	0.360	RXRA	0.589	SIN3AK20	0.378	SMARCA4	0.612	SPI1	0.149	ZNF143	-0.606	SMC3	-0.141	REST	-0.147
RFX5	0.653	SETDB1	0.292	SIRT6	0.430	SMARCB1	0.735	SRF	0.285	ZNF263	0.273	SP1	-0.346	RFX5	-0.030
RPC155	0.666	SIN3AK20	0.678	SIX5	0.169	SMARCC1	0.476	STAT1	0.246			SPI1	-0.203	RPC155	-0.066
RXRA	0.567	SIRT6	0.639	SMARCA4	0.216	SMC3	0.509	STAT2	0.326			SREBP1	-0.559	RXRA	-0.028
SETDB1	0.247	SIX5	0.689	SMARCB1	0.425	SP1	0.649	STAT3	0.114			SRF	-0.385	SETDB1	-0.229
SIN3AK20	0.637	SMARCA4	0.518	SMARCC1	0.463	SPI1	0.468	SUZ12	0.085			STAT1	-0.051	SIN3AK20	-0.024
SIRT6	0.409	SMARCB1	0.784	SMARCC2	0.543	SRF	0.614	TAF1	0.224			STAT2	-0.195	SIRT6	-0.194
SIX5	0.516	SMARCC1	0.688	SMC3	0.320	STAT1	0.573	TAF7	0.580			STAT3	0.012	SIX5	-0.012
SMARCA4	0.269	SMC3	0.502	SP1	0.290	STAT2	0.633	TAL1	0.113			SUZ12	-0.043	SMARCA4	0.018
SMARCB1	0.497	SP1	0.622	SP2	0.104	STAT3	0.275	TBP	0.367			TAF1	-0.216	SMARCB1	-0.136
SMARCC1	0.674	SP2	0.559	SPI1	0.310	SUZ12	0.523	TCF12	0.432			TAF7	-0.193	SMARCC1	-0.210
SMARCC2	0.691	SPI1	0.492	SRF	0.254	TAF1	0.631	TCF7L2	0.235			TAL1	-0.155	SMARCC2	-0.077

SMC3	0.417
SP1	0.558
SPI1	0.412
SRF	0.431
STAT1	0.368
STAT2	0.443
STAT3	0.354
SUZ12	0.303
TAF1	0.587
TAF7	0.681
TAL1	0.389
TBP	0.592
TCF12	0.500
TCF7L2	0.428
TFAP2A	0.545
TFAP2C	0.524
THAP1	0.552
USF1	0.561
USF2	0.631
WRNIP1	0.546
YY1	0.539
ZBTB33	0.554
ZBTB7A	0.591
ZEB1	0.506
ZNF143	0.477
ZNF263	0.524
ZNF274	0.164
ZZZ3	0.375

SRF	0.546
STAT1	0.524
STAT2	0.571
STAT3	0.479
SUZ12	0.739
TAF1	0.644
TAF7	0.622
TAL1	0.517
TBP	0.645
TCF12	0.579
TCF7L2	0.562
TFAP2A	0.615
TFAP2C	0.625
USF1	0.586
USF2	0.590
WRNIP1	0.501
YY1	0.616
ZBTB33	0.477
ZBTB7A	0.710
ZEB1	0.559
ZNF143	0.564
ZNF263	0.547
ZNF274	0.312
ZZZ3	0.362

STAT1	0.344
STAT3	0.306
SUZ12	-0.302
TAF1	0.261
TAF7	0.106
TAL1	0.356
TBP	0.356
TCF12	0.335
TCF7L2	0.404
TFAP2A	0.340
TFAP2C	0.326
THAP1	0.223
USF1	0.317
USF2	0.304
YY1	0.279
ZBTB33	0.314
ZBTB7A	0.303
ZEB1	0.252
ZNF143	0.296
ZNF263	0.292
ZNF274	0.590
ZZZ3	-0.094

TAF7	0.791
TAL1	0.401
TBP	0.630
TCF12	0.516
TCF7L2	0.353
TFAP2A	0.575
TFAP2C	0.511
USF1	0.589
USF2	0.673
YY1	0.609
ZBTB33	0.657
ZBTB7A	0.798
ZEB1	0.636
ZNF143	0.693
ZNF263	0.491
ZNF274	0.217
ZZZ3	-0.300

TFAP2A	-0.019
TFAP2C	-0.011
USF1	0.285
USF2	0.308
YY1	0.164
ZBTB7A	0.199
ZEB1	0.292
ZNF143	0.236
ZNF263	0.227
ZNF274	0.668
ZZZ3	0.535

TBP	-0.199
TCF12	-0.264
TCF7L2	-0.110
TFAP2A	-0.152
TFAP2C	-0.134
USF1	-0.393
USF2	-0.422
YY1	-0.218
ZBTB33	-0.489
ZBTB7A	-0.468
ZEB1	-0.419
ZNF143	-0.179
ZNF263	-0.542
ZNF274	-0.857
ZZZ3	0.310

SMC3	0.032
SP1	-0.095
SP2	-0.109
SPI1	-0.059
SREBP1	-0.205
SRF	-0.061
STAT1	-0.132
STAT2	-0.015
STAT3	0.062
SUZ12	-0.390
TAF1	0.035
TAF7	-0.060
TAL1	-0.092
TBP	-0.010
TCF12	-0.073
TCF7L2	-0.110
TFAP2A	-0.125
TFAP2C	-0.124
USF1	-0.172
USF2	-0.171
YY1	0.016
ZBTB33	-0.131
ZBTB7A	-0.143
ZEB1	-0.072
ZNF143	0.041
ZNF263	-0.189
ZNF274	-0.339
ZZZ3	0.199
