# Supplementary material for: Functional chromatin features are associated with structural mutations in cancer
Source: BMC Genomics. 2014 Nov 23;15(1):1013. doi: 10.1186/1471-2164-15-1013 (PMC4253614; doi:10.1186/1471-2164-15-1013)
Supplement: Supplementary file 4 — Additional file 4: Histogram of chromatin state at the breakpoints in three different SM callsets. Teal and white bars indicate observed vs. expected values, respectively. Erros bars indicate binomial standard error. Left panels show the full histograms, the right panels show respective zoom-in views at low frequency. (PDF 1 MB) [file 12864_2014_6709_MOESM4_ESM.pdf]

Additional file 4 Broad-GM12878

A1 Breast-Inaki

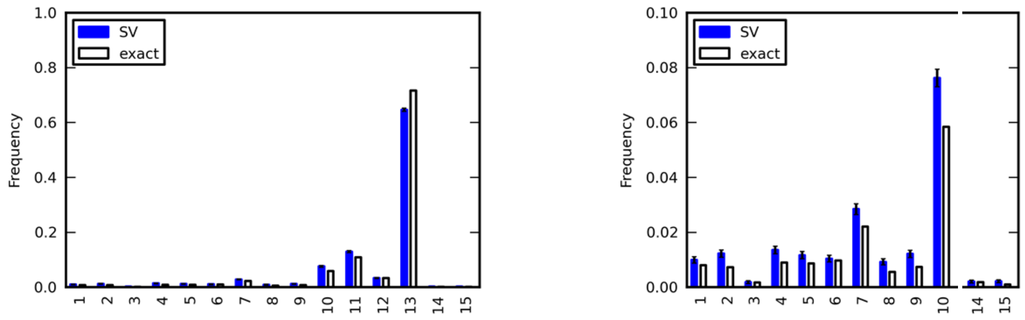

A2 Breast-NikZainal

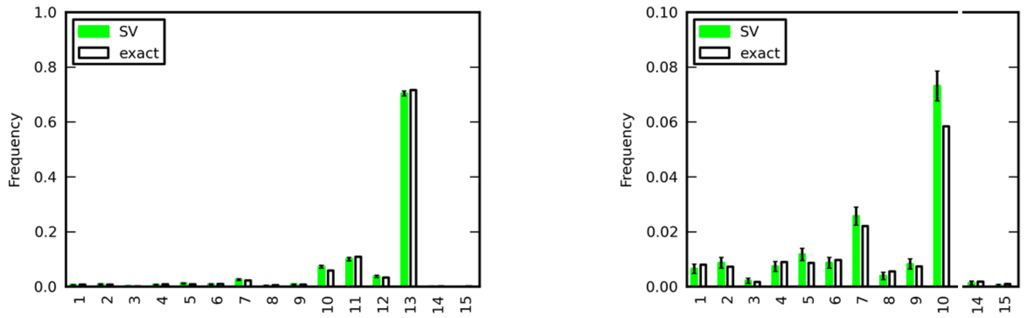

A3 Ovarian-McBride

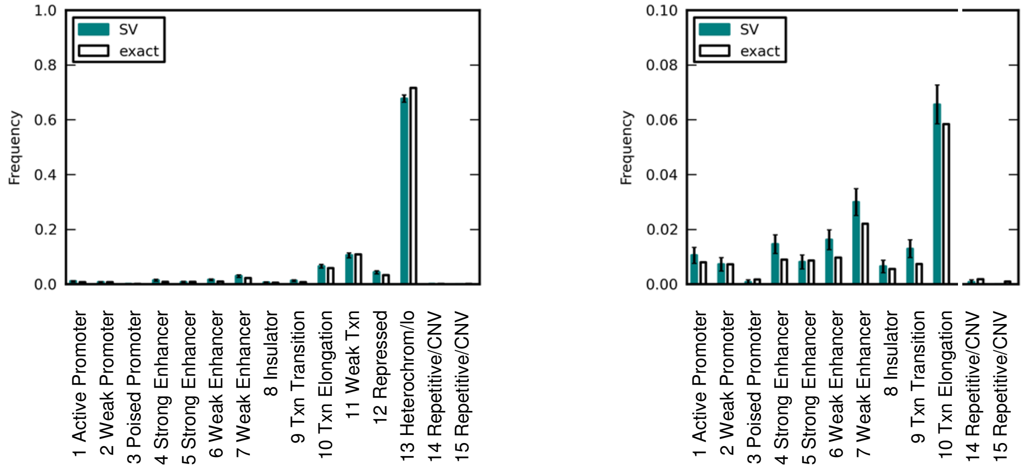

Histogram of chromatin state at the breakpoints in three different SM callsets. Teal and white bars indicate observed vs. expected values, respectively. Erros bars indicate binomial standard error. Left panels show the full histograms, the right panels show respective zoom-in views at low frequency.

Additional file 4 Broad-H1HESC

B1

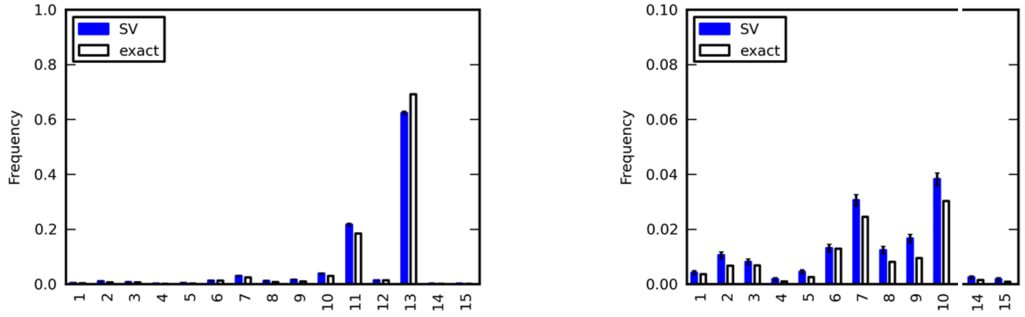

B2

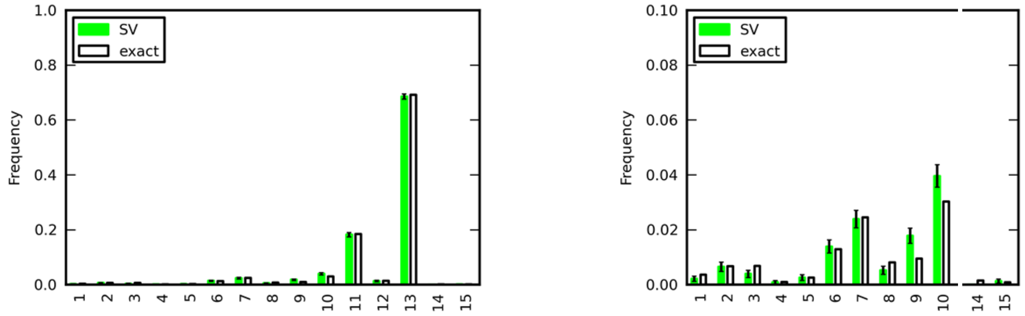

B3

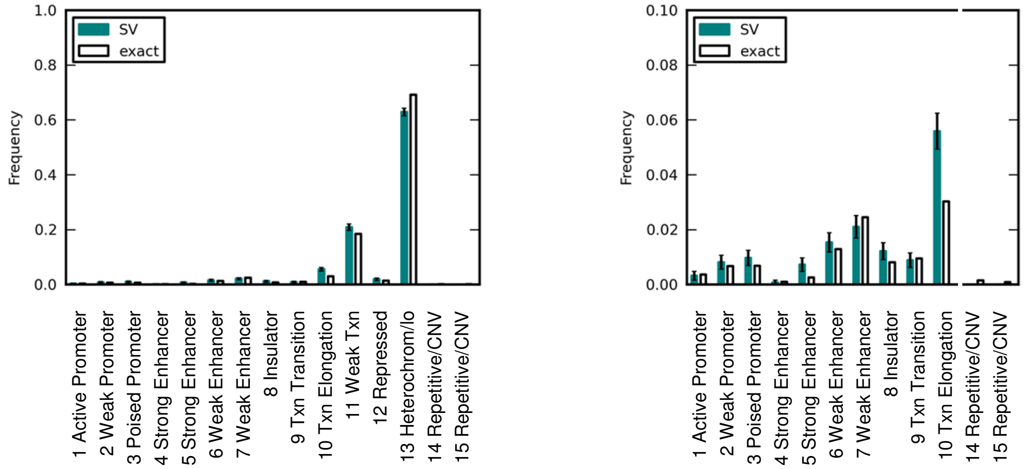

Histogram of chromatin state at the breakpoints in three different SM callsets. Teal and white bars indicate observed vs. expected values, respectively. Erros bars indicate binomial standard error. Left panels show the full histograms, the right panels show respective zoom-in views at low frequency.

Additional file 4 Broad-HepG2

C1

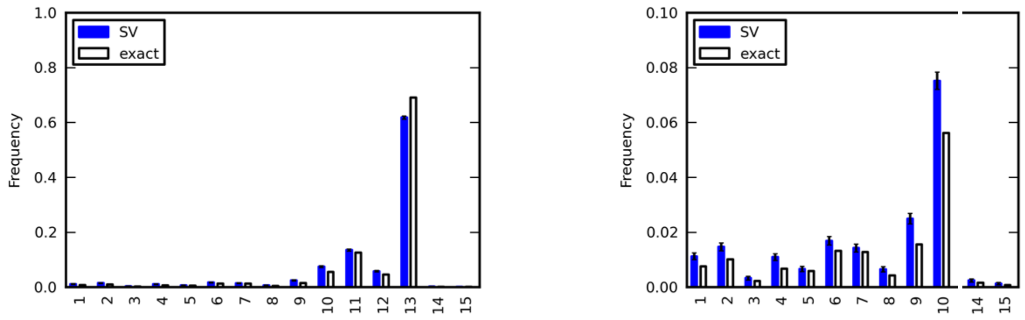

C2

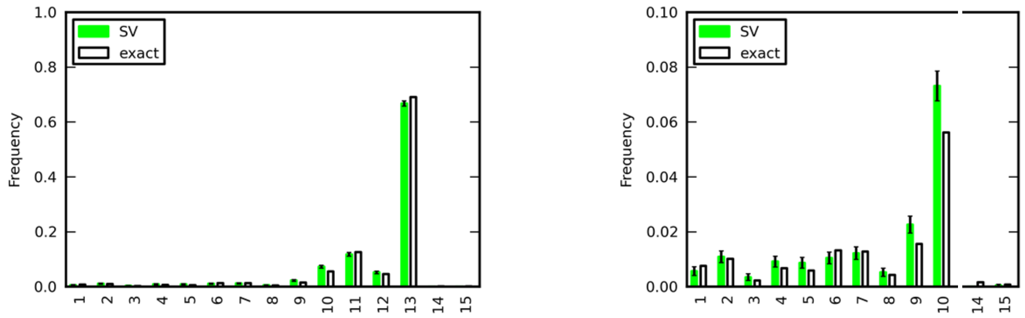

C3

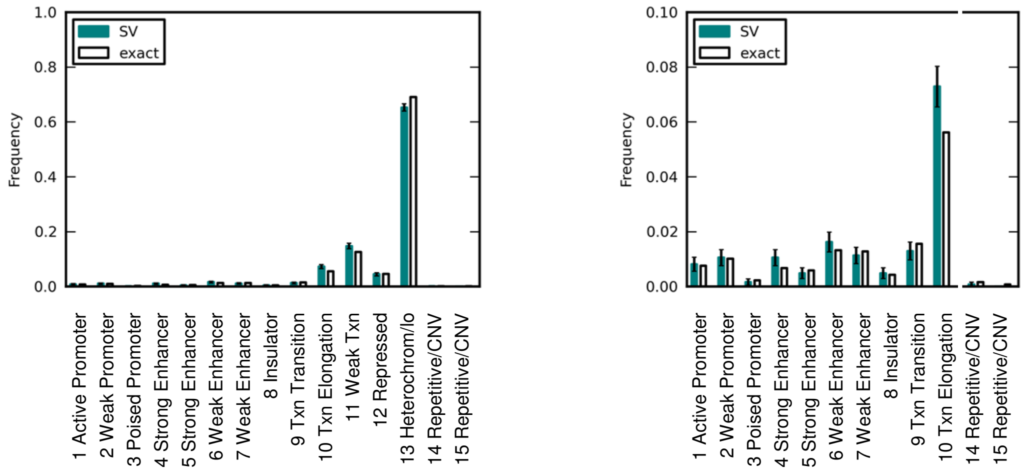

Histogram of chromatin state at the breakpoints in three different SM callsets. Teal and white bars indicate observed vs. expected values, respectively. Erros bars indicate binomial standard error. Left panels show the full histograms, the right panels show respective zoom-in views at low frequency.

Additional file 4 Broad-HMEC

D1

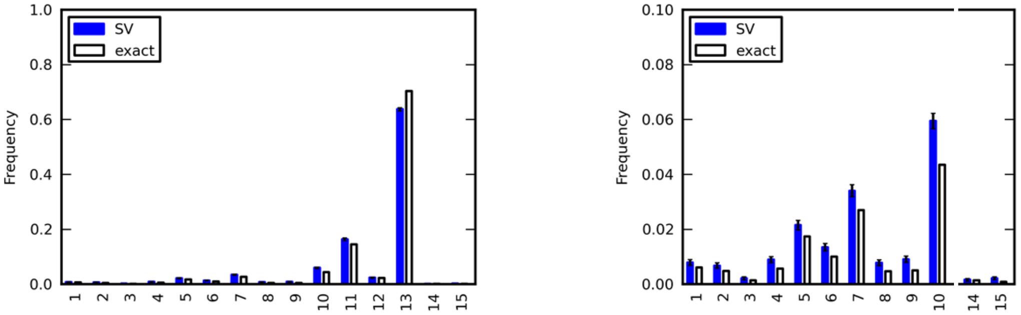

D2

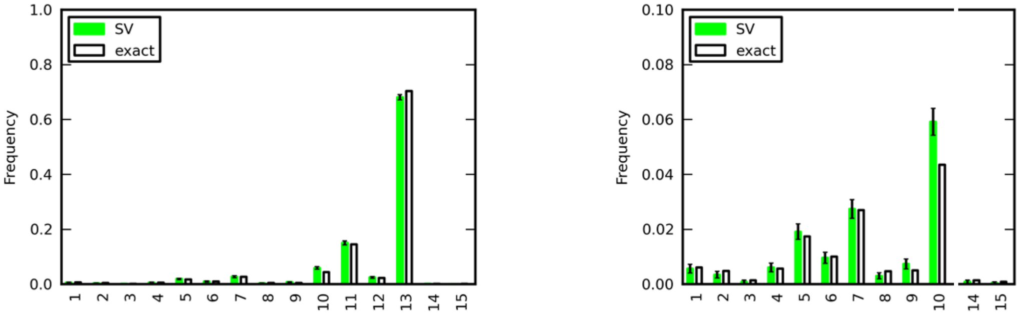

D3

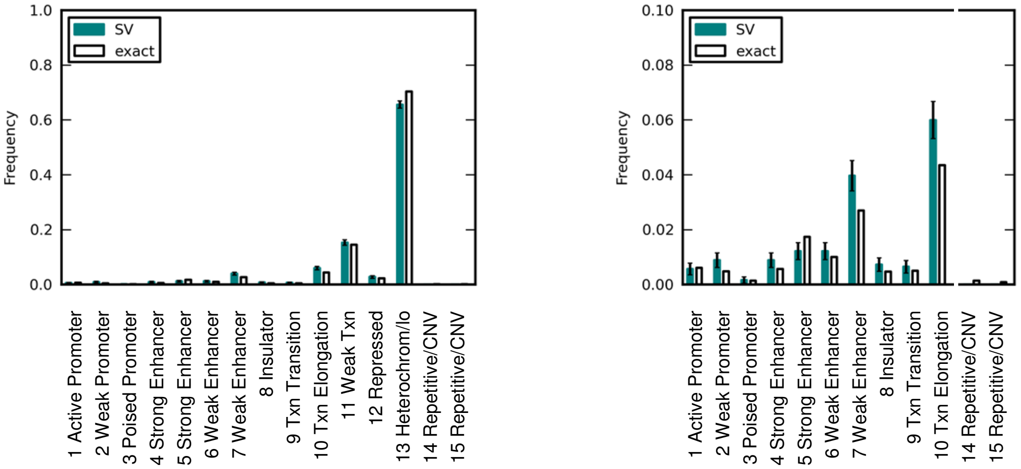

Histogram of chromatin state at the breakpoints in three different SM callsets. Teal and white bars indicate observed vs. expected values, respectively. Erros bars indicate binomial standard error. Left panels show the full histograms, the right panels show respective zoom-in views at low frequency.

Additional file 4

Broad-HSMM

E1

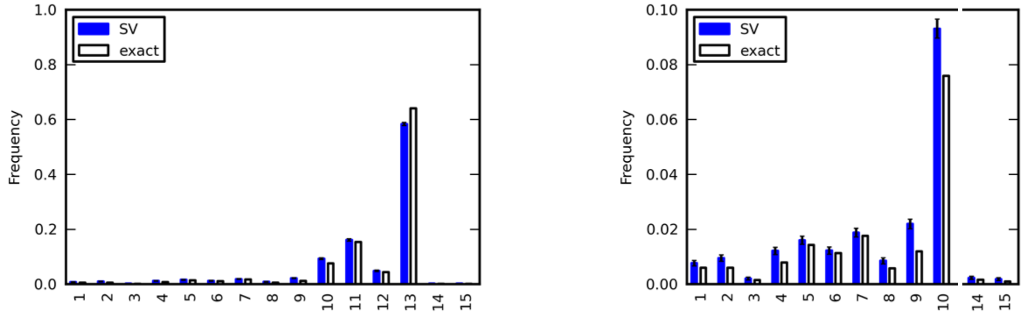

E2

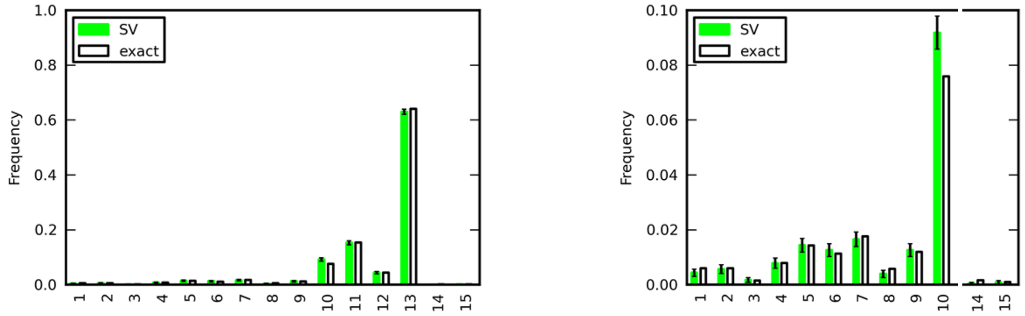

E3

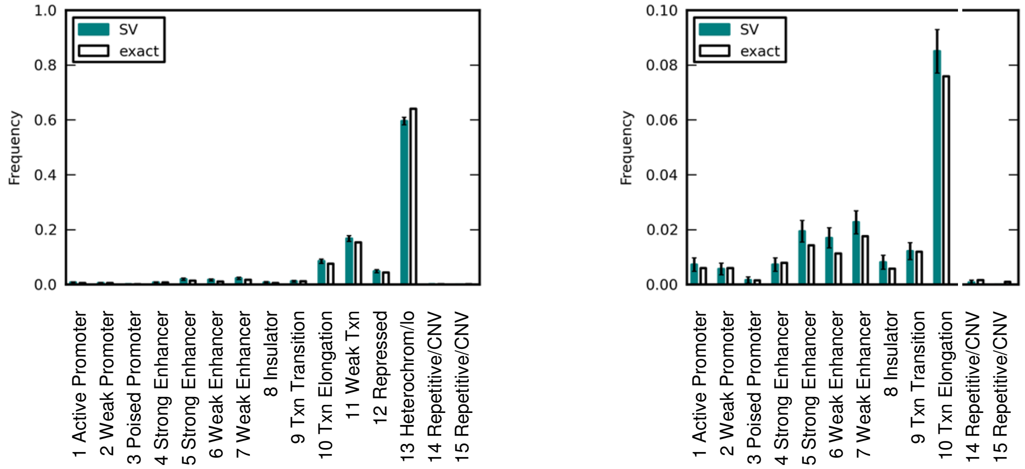

Histogram of chromatin state at the breakpoints in three different SM callsets. Teal and white bars indicate observed vs. expected values, respectively. Erros bars indicate binomial standard error. Left panels show the full histograms, the right panels show respective zoom-in views at low frequency.

Additional file 4 Broad-HUVEC

F1

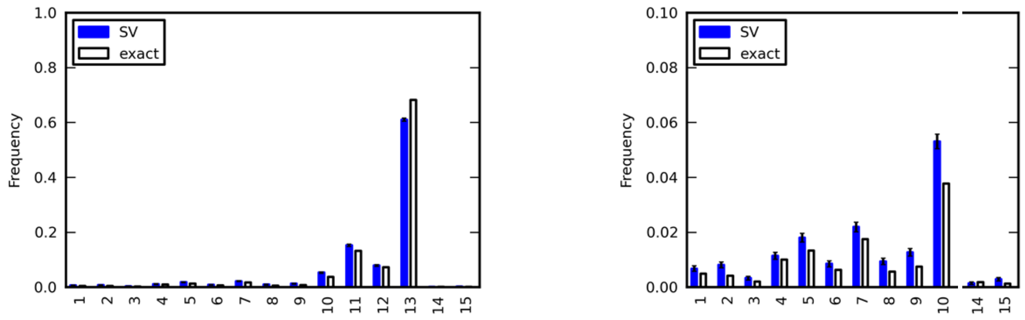

F2

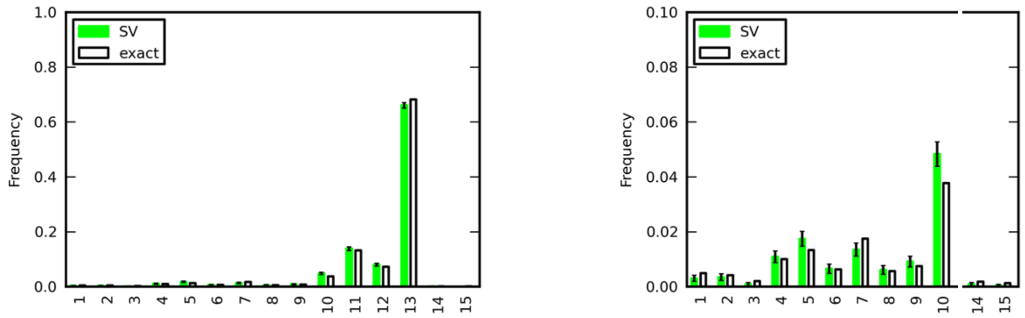

F3

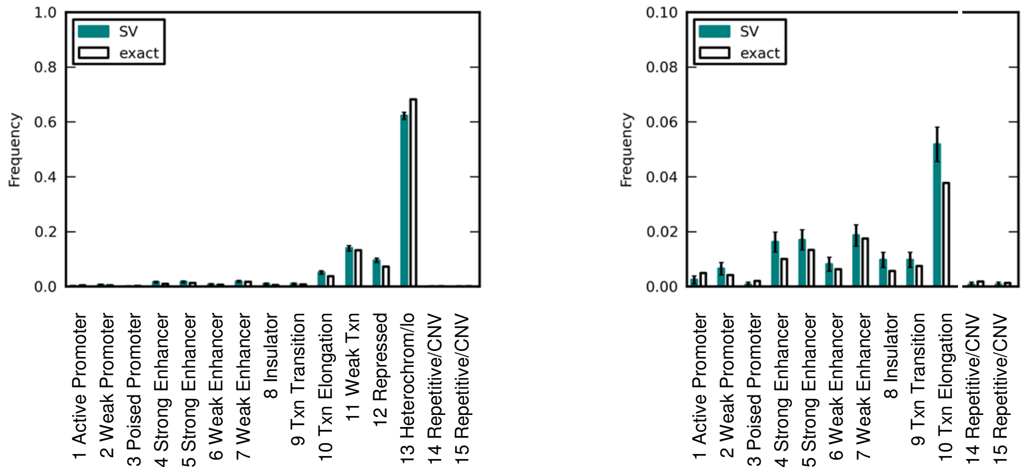

Histogram of chromatin state at the breakpoints in three different SM callsets. Teal and white bars indicate observed vs. expected values, respectively. Erros bars indicate binomial standard error. Left panels show the full histograms, the right panels show respective zoom-in views at low frequency.

Additional file 4 **Broad-K562**

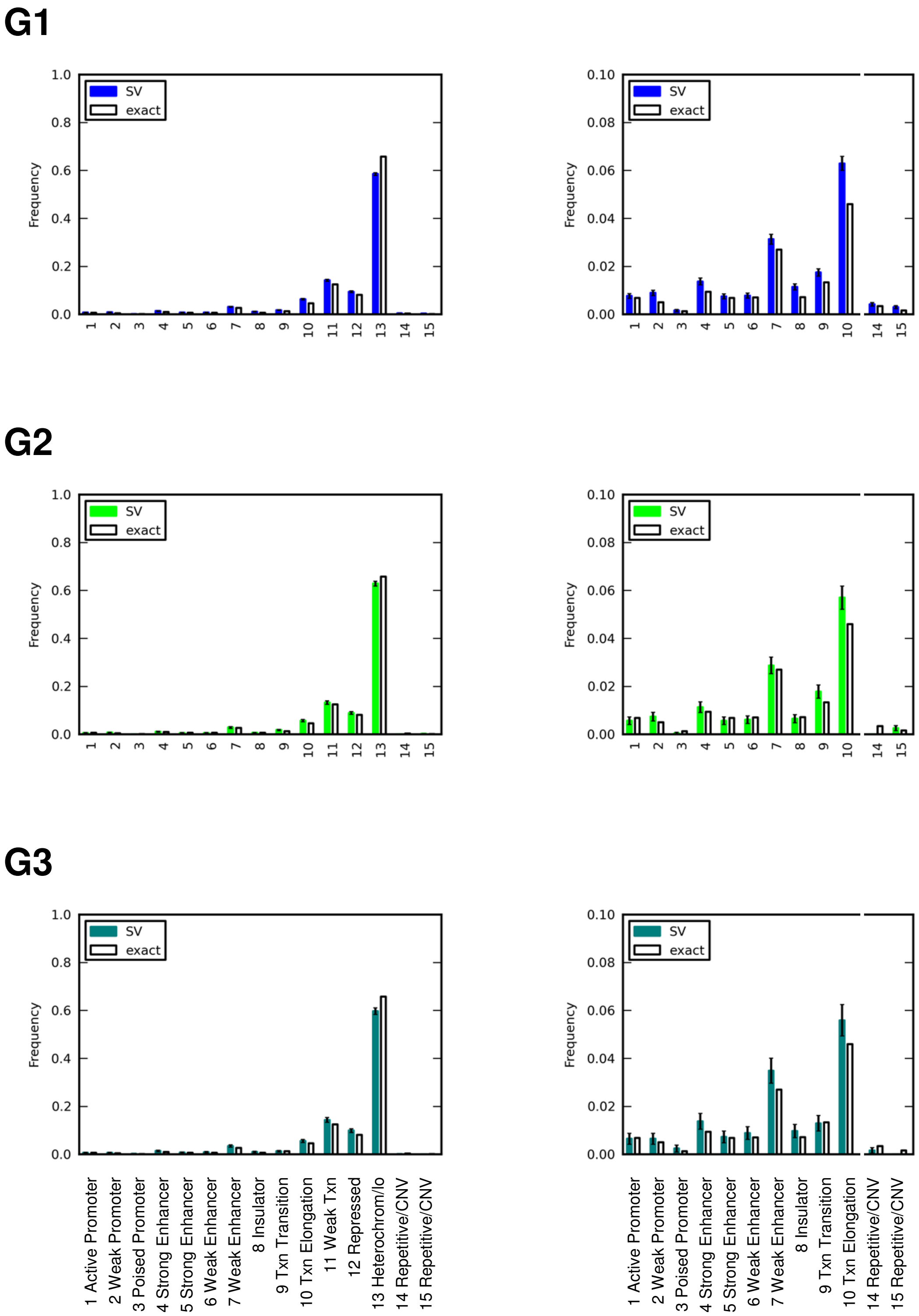

Histogram of chromatin state at the breakpoints in three different SM callsets. Teal and white bars indicate observed vs. expected values, respectively. Erros bars indicate binomial standard error. Left panels show the full histograms, the right panels show respective zoom-in views at low frequency.

Additional file 4 Broad-NHEK

H1

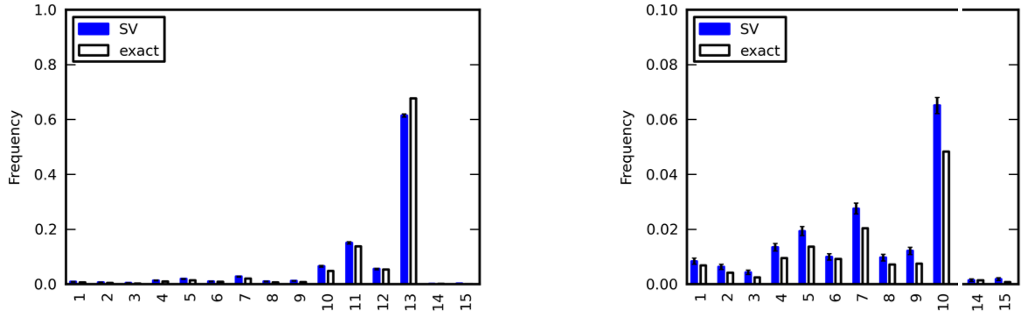

H2

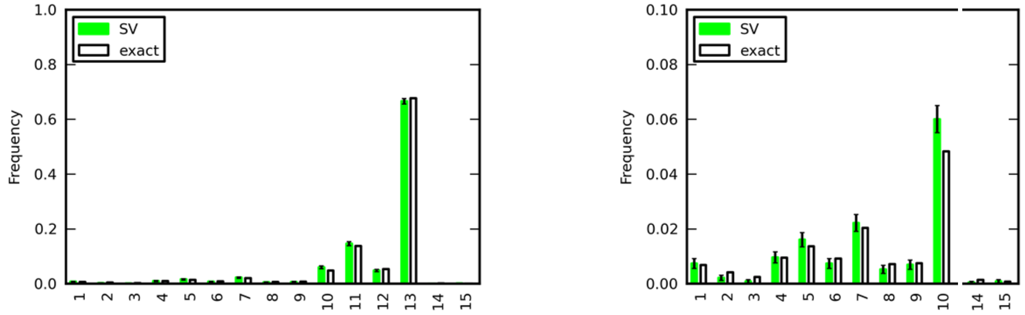

H3

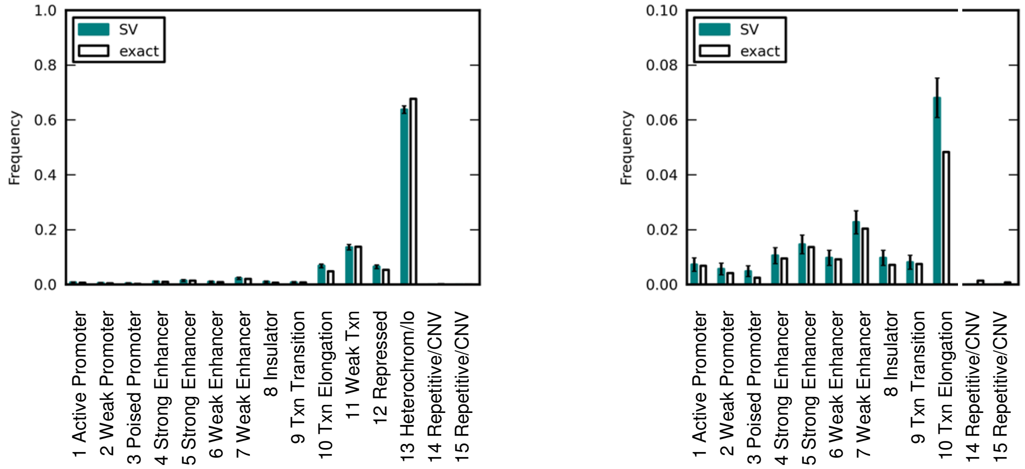

Histogram of chromatin state at the breakpoints in three different SM callsets. Teal and white bars indicate observed vs. expected values, respectively. Erros bars indicate binomial standard error. Left panels show the full histograms, the right panels show respective zoom-in views at low frequency.

Additional file 4   Broad-NHLF

I1

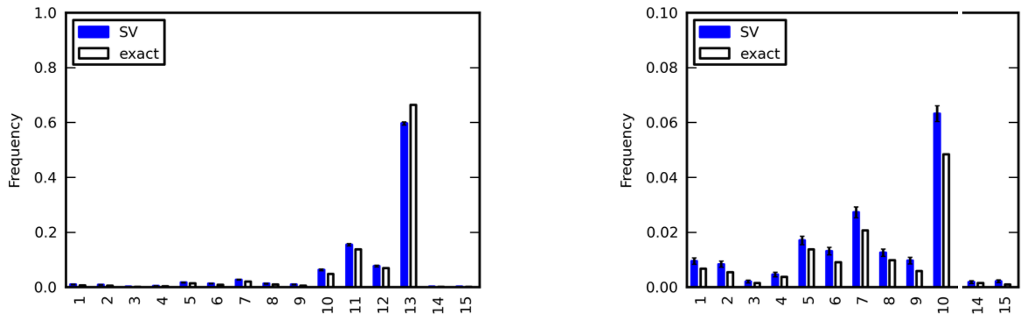

I2

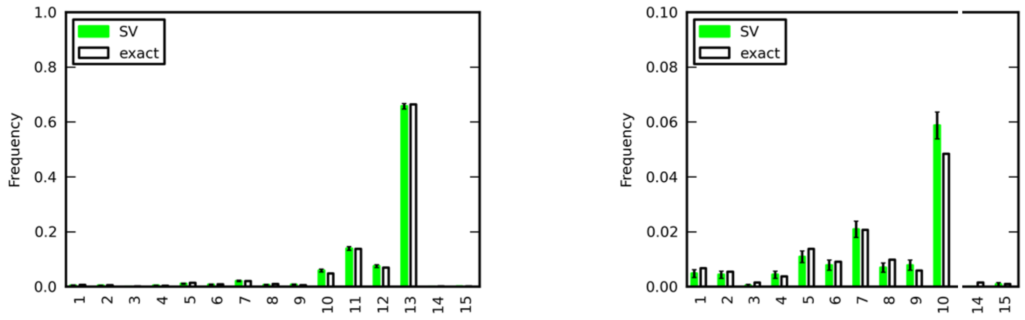

I3

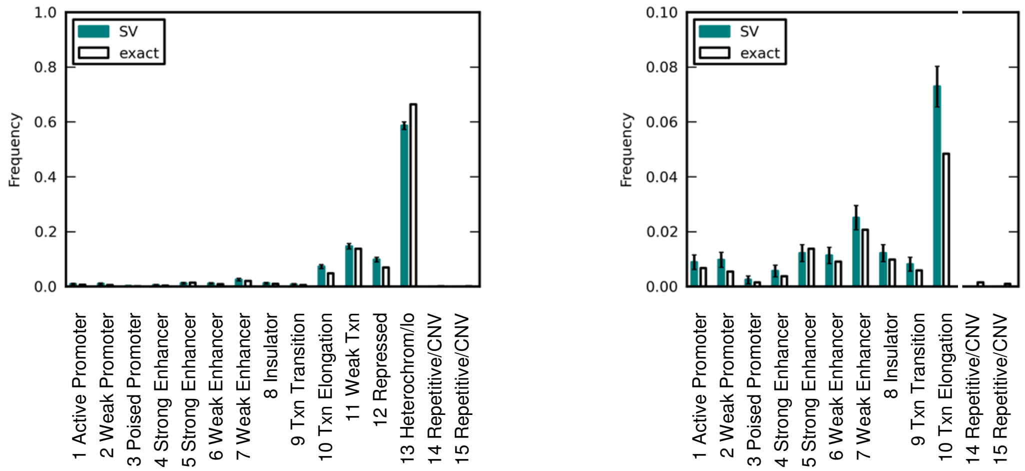

Histogram of chromatin state at the breakpoints in three different SM callsets. Teal and white bars indicate observed vs. expected values, respectively. Erros bars indicate binomial standard error. Left panels show the full histograms, the right panels show respective zoom-in views at low frequency.
