# Supplementary material for: Functional chromatin features are associated with structural mutations in cancer
Source: BMC Genomics. 2014 Nov 23;15(1):1013. doi: 10.1186/1471-2164-15-1013 (PMC4253614; doi:10.1186/1471-2164-15-1013)
Supplement: Supplementary file 5 — Additional file 5: Patterns of ChIP-seq enrichment extend up to 200 kb. Odds ratio values across all available protein binding ChIP-seq experiments. Each point represents a different protein ChIP-seq experiment, with odds ratio calculated separately near (≤10 kb) genes (horizontal axis) and far from (>10 kb) genes (vertical axis). Positive values indicate enrichment of protein ChIP-seq signal within 200 kb of SM breakpoints. Data shown in various SM callsets: Breast-Inaki (A), Breast-Stephens (B), Breast-NikZainal (C), Ovarian-McBride (D), Colorectal-Bass (E), Head&Neck-Stransky (F), Prostate-Berger (G), Prostate-Baca (H). (PDF 351 KB) [file 12864_2014_6709_MOESM5_ESM.pdf]

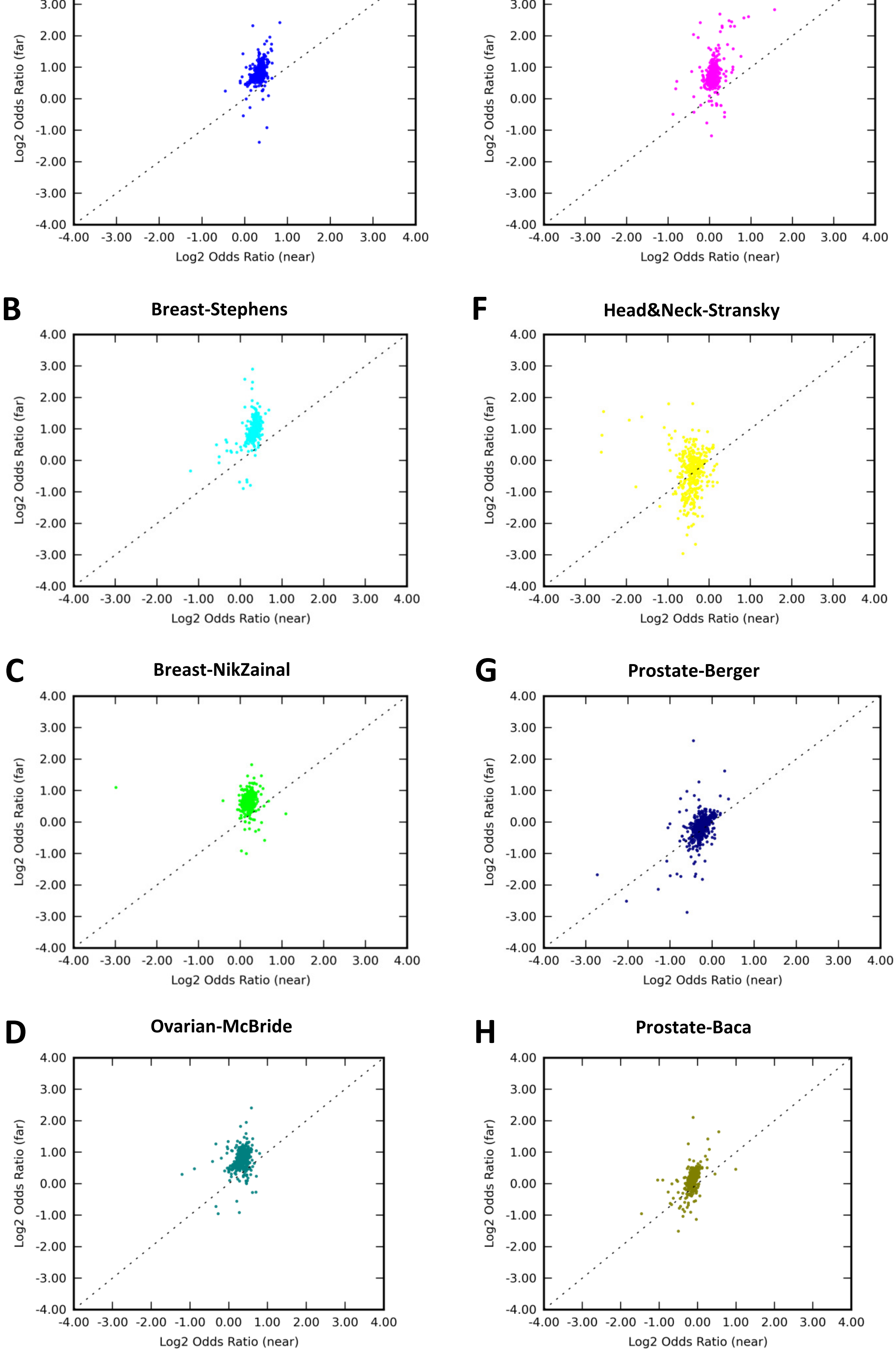

Patterns of ChIP-seq enrichment extend up to 200kb, even in the callsets with the least obvious enrichemnt pattern. In Prostate-Berger the average log2-odds in the far region (-0.17 ± 0.46) is still somewhat larger than that in the near region (-0.27 ± 0.24) and in Head&Neck-Stransky the y>x behavior is still observable though of modest effect (-0.37 ± 0.70 far region; -0.41 ± 0.30 near region). Odds ratio values across all available protein binding ChIP-seq experiments. Each point represents a different protein ChIP-seq experiment, with odds ratio calculated separately near (<10 kb) genes (horizontal axis) and far from (>10 kb) genes
