# Supplementary figures and images for: Functional chromatin features are associated with structural mutations in cancer
Source: BMC Genomics. 2014 Nov 23;15(1):1013. doi: 10.1186/1471-2164-15-1013 (PMC4253614; doi:10.1186/1471-2164-15-1013)

A

Breast-Stephens

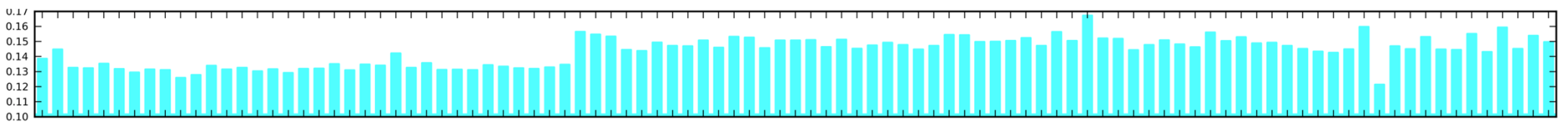

B

Ovarian-McBride

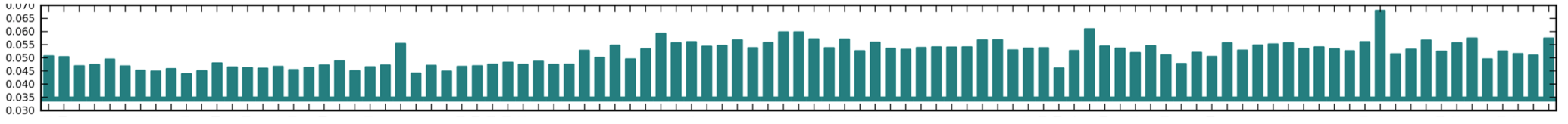

C

ETS+/CHD1wt

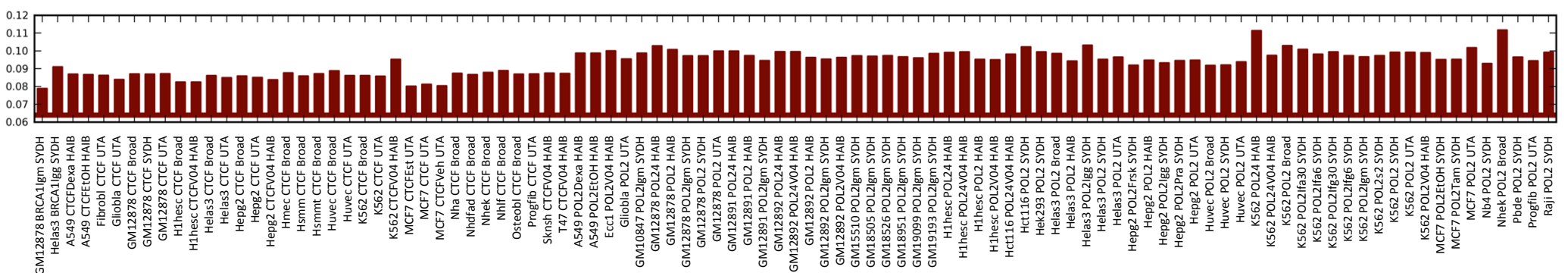

Supplement: Supplementary file 6 — Additional file 6: Enrichment of protein binding events in the vicinity of breakpoints is common across proteins with diverse functions, such as transcription (eg. Pol II), DNA repair (BRCA1) and 3D structure (CTCF). Bars indicates fraction of ChIP-seq signal falling within 50 kb of any breakpoint. The horizontal line indicates baseline expectations, i.e. the fraction of the genome falling within that distance of any breakpoint. (PDF 205 KB) [file 12864_2014_6709_MOESM6_ESM.pdf]
