# Supplementary material for: Functional chromatin features are associated with structural mutations in cancer
Source: BMC Genomics. 2014 Nov 23;15(1):1013. doi: 10.1186/1471-2164-15-1013 (PMC4253614; doi:10.1186/1471-2164-15-1013)
Supplement: Supplementary file 7 — Additional file 7: Effect of cell type on enrichment of protein binding ChIP-seq signal. Each point represents a different protein binding ChIP-seq experiment, with odds ratio calculated separately near genes (horizontal axis) and far from genes (vertical axis). Positive values indicate enrichment of protein ChIP-seq signal within 50 kb of SM breakpoints. Experiments performed in the stem cell lines are shown in the top row, in the cancer cell lines in the middle and in the EBV-transformed lymphoblastoid cell lines in the bottom row. Δ indicates the difference of log OR between the regions near and far from genes, i.e. the average location of the cloud of points above the diagonal line, averaged over n experiments. Data shown in various SM callsets: Breast-Inaki (A), Breast-Stephens (B), Breast-NikZainal (C), Ovarian-McBride (D), Colorectal-Bass (E), Head&Neck-Stransky (F), Prostate-Berger (G), Prostate-Baca (H). (PDF 392 KB) [file 12864_2014_6709_MOESM7_ESM.pdf]

Additional File 7

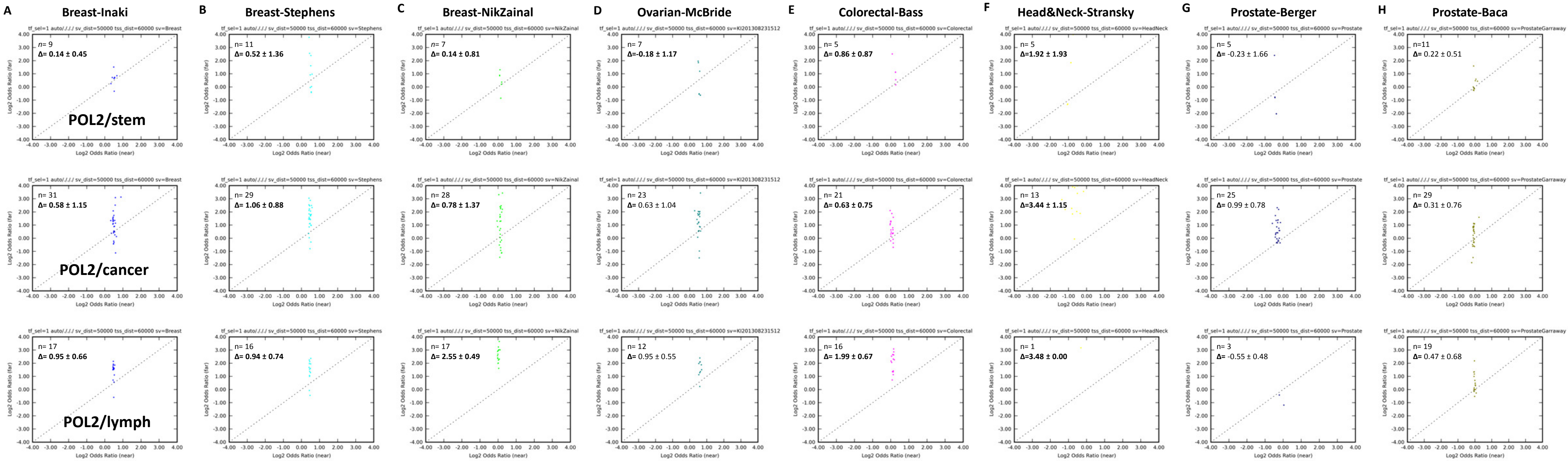

Effect of cell type on enrichment of protein binding ChIP-seq signal. Each point represents a different protein binding ChIP-seq experiment, with odds ratio calculated separately near genes (horizontal axis) and far from genes (vertical axis). Positive values indicate enrichment of protein ChIP-seq signal within 50 kb of SM breakpoints. Experiments performed in the stem cell lines are shown in the top row, in the cancer cell lines in the middle and in the EBV-transformed lymphoblastoid cell lines in the bottom row. **Δ** indicates the difference of log OR between the regions near and far from genes, i.e. the average location of the cloud of points above the diagonal line, averaged over *n* experiments. Data shown in various SM callsets: Breast-Inaki (A), Breast-Stephens (B), Breast-NikZainal (C), Ovarian-McBride (D), Colorectal-Bass (E), Head&Neck-Stransky (F), Prostate-Berger (G), Prostate-Baca (H).
