# Supplementary material for: Functional chromatin features are associated with structural mutations in cancer
Source: BMC Genomics. 2014 Nov 23;15(1):1013. doi: 10.1186/1471-2164-15-1013 (PMC4253614; doi:10.1186/1471-2164-15-1013)
Supplement: Supplementary file 8 — Additional file 8: Effect of SM calling pipeline. Odds ratio values across all available protein binding ChIP-seq experiments. Each point represents a different protein binding ChIP-seq experiment, with odds ratio calculated separately near (≤60 kb) genes (horizontal axis) and far from (>60 kb) genes (vertical axis). Positive values indicate enrichment of protein ChIP-seq signal within 50 kb of SM breakpoints. Data shown in two cancers from The Cancer Genome Atlas (breast cancer “BRCA” in the top row and lung cancer “LUSC” in the bottom row) using two different SM callers (Hydra on the left and Meerkat on the right). (PDF 97 KB) [file 12864_2014_6709_MOESM8_ESM.pdf]

Additional File 8

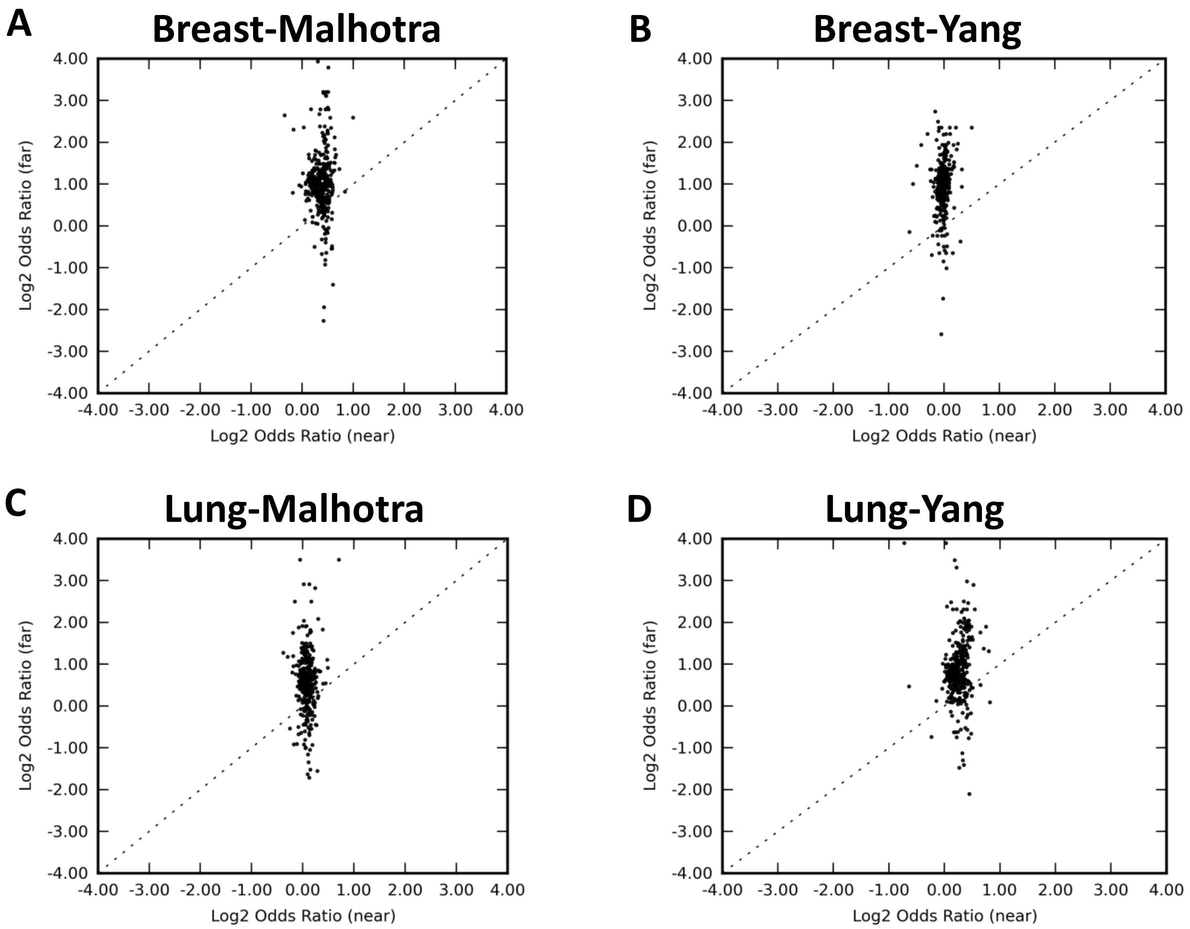

Effect of SM calling pipeline. Odds ratio values across all available protein binding ChIP-seq experiments. Each point represents a different protein binding ChIP-seq experiment, with odds ratio calculated separately near (≤60 kb) genes (horizontal axis) and far from (>60 kb) genes (vertical axis). Positive values indicate enrichment of protein ChIP-seq signal within 50 kb of SM breakpoints. Data shown in two cancers from The Cancer Genome Atlas (breast cancer “BRCA” in the top row and lung cancer “LUSC” in the bottom row) using two different SM callers (Hydra on the left and Meerkat on the right).
